# The outcomes measured and reported in intracranial meningioma clinical trials: A systematic review

**DOI:** 10.1093/noajnl/vdae030

**Published:** 2024-03-02

**Authors:** Christopher P Millward, Sumirat M Keshwara, Terri S Armstrong, Heather Barrington, Sabrina Bell, Andrew R Brodbelt, Helen Bulbeck, Linda Dirven, Paul L Grundy, Abdurrahman I Islim, Mohsen Javadpour, Shelli D Koszdin, Anthony G Marson, Michael W McDermott, Torstein R Meling, Kathy Oliver, Puneet Plaha, Matthias Preusser, Thomas Santarius, Nisaharan Srikandarajah, Martin J B Taphoorn, Carole Turner, Colin Watts, Michael Weller, Paula R Williamson, Gelareh Zadeh, Amir H Zamanipoor Najafabadi, Michael D Jenkinson, EORTC BTG, ICOM, EANO, SNO, RANO-PRO, BNOS, SBNS, BIMS, TBTC, International Brain Tumour Alliance, Brainstrust, and Brain Tumour Foundation of Canada, Kenneth Aldape, Abdurrahman I Islim, Karolyn Au, Jill Barnhartz-Sloan, Wenya Linda Bi, Felix Behling, Priscilla K Brastianos, Chaya Brodie, Nicholas Butowski, Carlos Carlotti, Ana Castro, Aaron Cohen-Gadol, Marta Couce, Michael D Cusimano, Francesco DiMeco, Katharine Drummond, Ian F Dunn, Craig Erker, Michelle Felicella, Daniel M Fountain, Evanthia Galanis, Norbert Galldiks, Caterina Giannini, Roland Goldbrunner, Brent Griffith, Rintaro Hashizume, C Oliver Hanemann, Christel Herold-Mende, Luke Hnenny, Craig Horbinski, Raymond Y Huang, David James, Michael D Jenkinson, Christine Jungk, Gerhard Jungwirth, Timothy J Kaufmann, Boris Krischek, Sylvia Kurz, Daniel Lachance, Christian Lafougère, Katrin Lamszus, Ian Lee, Jeff C Liu, Serge Makarenko, Tathiana Malta, Yasin Mamatjan, Alireza Mansouri, Christian Mawrin, Michael McDermott, Christopher P Millward, Jennifer Moliterno-Gunel, Andrew Morokoff, David Munoz, Farshad Nassiri, Houtan Noushmehr, Ho-Keung Ng, Arie Perry, Farhad Pirouzmand, Laila M Poisson, Bianca Pollo, Aditya Ragunathan, David R Raleigh, Mirjam Renovanz, Franz Ricklefs, Felix Sahm, Andrea Saladino, Antonio Santacroce, Thomas Santarius, Jens Schittenhelm, Christian Schichor, David Schultz, Nils O Schmidt, Warren Selman, Helen Shih, Andrew Sloan, Julian Spears, Matija Snuderl, James Snyder, Suganth Suppiah, Erik Sulman, Ghazaleh Tabatabai, Marcos Tatagiba, Marco Timmer, Daniela Tirapelli, Joerg C Tonn, Derek Tsang, Michael A Vogelbaum, Andreas von Deimling, Tobias Walbert, Simon Walling, Justin Z Wang, Patrick Y Wen, Manfred Westphal, Adriana M Workewych, Stephen Yip, Gabriel Zada, Gelareh Zadeh, Viktor Zherebitskiy

**Affiliations:** Institute of Systems, Molecular, and Integrative Biology, University of Liverpool, UK; Department of Neurosurgery, The Walton Centre NHS Foundation Trust, Liverpool, UK; Institute of Systems, Molecular, and Integrative Biology, University of Liverpool, UK; Department of Neurosurgery, The Walton Centre NHS Foundation Trust, Liverpool, UK; Neuro-Oncology Branch, Center for Cancer Research, National Cancer Institute, Bethesda, Maryland, USA; Institute of Population Health, University of Liverpool, UK; The Brain Tumour Charity, Hampshire, UK; Institute of Systems, Molecular, and Integrative Biology, University of Liverpool, UK; Department of Neurosurgery, The Walton Centre NHS Foundation Trust, Liverpool, UK; Brainstrust–The Brain Cancer People, Isle of Wight, UK; Department of Neurology, Leiden University Medical Center, Leiden, The Netherlands; Department of Neurology, Haaglanden Medical Center, The Hague, The Netherlands; Department of Neurosurgery, University Hospital Southampton, Southampton, UK; Institute of Systems, Molecular, and Integrative Biology, University of Liverpool, UK; Department of Neurosurgery, The Walton Centre NHS Foundation Trust, Liverpool, UK; National Centre for Neurosurgery, Beaumont Hospital, Dublin, Ireland; Veterans Affairs Healthcare System, Palo Alto, California, USA; Institute of Systems, Molecular, and Integrative Biology, University of Liverpool, UK; Department of Neurology, The Walton Centre NHS Foundation Trust, Liverpool, UK; Division of Neuroscience, Florida International University, Miami, Florida, USA; Department of Neurosurgery, Copenhagen University Hospital, Copenhagen, Denmark; International Brain Tumour Alliance, Tadworth, UK (K.O.).; Nuffield Department of Surgical Sciences, University of Oxford, Oxford, UK; Division of Oncology, Department of Medicine, Medical University of Vienna, Vienna, Austria; Department of Neurosurgery, Addenbrooke’s Hospital & University of Cambridge, Cambridge, UK; Institute of Systems, Molecular, and Integrative Biology, University of Liverpool, UK; Department of Neurology, Leiden University Medical Center, Leiden, The Netherlands; Department of Neurology, Haaglanden Medical Center, The Hague, The Netherlands; Department of Neurosurgery, Addenbrooke’s Hospital & University of Cambridge, Cambridge, UK; Institute of Cancer and Genomic Sciences, University of Birmingham, Birmingham, UK; Department of Neurology, University Hospital and University of Zurich, Zurich, Switzerland; Institute of Population Health, University of Liverpool, UK; Department of Surgery, University of Toronto, Toronto, Canada; Department of Ophthalmology, Leiden University Medical Centre, Haaglanden Medical Center, Haga Teaching Hospitals, Leiden, The Netherlands; Institute of Systems, Molecular, and Integrative Biology, University of Liverpool, UK; Department of Neurosurgery, The Walton Centre NHS Foundation Trust, Liverpool, UK

**Keywords:** clinical trial, core outcome set, COMET, meningioma, outcomes

## Abstract

**Background:**

Meningioma clinical trials have assessed interventions including surgery, radiotherapy, and pharmacotherapy. However, agreement does not exist on what, how, and when outcomes of interest should be measured. To do so would allow comparative analysis of similar trials. This systematic review aimed to summarize the outcomes measured and reported in meningioma clinical trials.

**Methods:**

Systematic literature and trial registry searches were performed to identify published and ongoing intracranial meningioma clinical trials (PubMed, Embase, Medline, CINAHL via EBSCO, and Web of Science, completed January 22, 2022). Reported outcomes were extracted verbatim, along with an associated definition and method of measurement if provided. Verbatim outcomes were deduplicated and the resulting unique outcomes were grouped under standardized outcome terms. These were classified using the taxonomy proposed by the “Core Outcome Measures in Effectiveness Trials” (COMET) initiative.

**Results:**

Thirty published articles and 18 ongoing studies were included, describing 47 unique clinical trials: Phase 2 *n* = 33, phase 3 *n* = 14. Common interventions included: Surgery *n* = 13, radiotherapy *n* = 8, and pharmacotherapy *n* = 20. In total, 659 verbatim outcomes were reported, of which 84 were defined. Following de-duplication, 415 unique verbatim outcomes remained and were grouped into 115 standardized outcome terms. These were classified using the COMET taxonomy into 29 outcome domains and 5 core areas.

**Conclusions:**

Outcome measurement across meningioma clinical trials is heterogeneous. The standardized outcome terms identified will be prioritized through an eDelphi survey and consensus meeting of key stakeholders (including patients), in order to develop a core outcome set for use in future meningioma clinical trials.

**Key Points**

1. Outcomes measured in meningioma clinical trials are highly heterogeneous.
2. One hundred and fifteen standardized outcome terms were created from 659 verbatim outcome terms that were extracted.
3. These will be prioritized through an eDelphi survey and consensus meeting to define a core outcome set.

**Importance of the Study**

Meningioma clinical trials have assessed interventions including surgery, radiotherapy, and pharmacotherapy. However, agreement does not exist on what, how, and when outcomes of interest should be measured. This prevents comparison of data from similar clinical trials. In this methodological review, we have systematically identified relevant meningioma clinical trials, extracted outcomes measured, and applied standardized outcome terms to those with similar meaning and context. The standardized outcome terms will be prioritized through an eDelphi survey and consensus meeting of key stakeholders (including patients) in a subsequent step. This novel approach paves the way for the development of a Core Outcome Set (COSMIC: Intervention) for use in future meningioma clinical trials. This work is one half of The COSMIC Project (Development of Core Outcome Sets for Meningioma in Clinical Studies).

Meningiomas account for 39% of all primary tumors of the central nervous system, and have an estimated age-adjusted incidence of 9.1 per 100 000 population per year, increasing to 57.3 per 100 000 in adults over the age of 85.^1^ They are more than twice as common in females (12.4 vs. 5.5 per 100 000 population), and the median age at diagnosis is 66 years.^1^ The World Health Organization (WHO) classification of tumors of the central nervous system describes 3 grades and fifteen histopathological subtypes of meningioma, with the latest version incorporating molecular markers for the first time.^2^ Distribution by WHO grade is currently as follows; 80.4% benign (WHO grade 1), 17.9% atypical (WHO grade 2), and 1.6% malignant (WHO grade 3).^2^

For meningiomas that cause symptoms, threaten neurovascular structures, or demonstrate interval growth on imaging, a treatment intervention is warranted, and surgical resection is usually the first-line management strategy, although stereotactic radiosurgery may be used for small tumors.^3,4^ For patients who are poor surgical candidates, or have inoperable, residual, or recurrent disease, radiotherapy may be used as either primary or adjuvant treatment. For all meningiomas, there exists a long-term risk of recurrence, and important research questions remain to be answered concerning the management of such patients. For grade 3 meningioma, recurrence is inevitable and surgery and radiotherapy options become exhausted over time.

Clinical trials for intracranial meningioma are uncommon, but have largely explored treatment options for patients with high-grade, recurrent, and progressive disease. For instance, 2 phase 2 studies have investigated the efficacy of adjuvant radiotherapy following surgical resection of high-grade meningioma; radiation therapy oncology group 0539^5^ and the European Organization for Research and Treatment of Cancer (EORTC) 22 042,^6^ and there are 2 phase 3 randomized controlled trials to be reported which aim to establish the role of adjuvant radiotherapy after gross-total resection of WHO grade 2 meningioma; ROAM/EORTC 1308^7^ and NRG-BN003.^8^ Despite multiple studies investigating a wide range of agents, no effective pharmacotherapy treatments have been identified, possibly due to the recruitment of heavily pretreated patients with heterogeneous pathology, treatment-resistant tumor cells, and limited knowledge of the disease biology.^3,4^ Most recently, Preusser et al.^9^ reported results for the first prospective randomized clinical trial for patients with grade 2 or 3 meningioma. The trial randomized patients to trabectedin (a tetrahydroisoquinoline alkaloid) versus local standard of care (physician’s choice). This multi-center study recruited 90 patients with more homogenous pathology over 22 months and provides the best evidence for progression-free survival (PFS) and overall survival (OS) in this patient population, and shows that prospective controlled trials are possible—despite the relative rarity of patients in each individual hospital.^10^

Over the past 5–10 years, genomic, transcriptomic, metabolomic, proteomic, and methylation profiling techniques have revealed the heterogeneity of meningioma, the limitations of the current WHO grading system for prognostication and impact this may have on clinical trial design and patient eligibility.^11–18^ This “meningiomics” revolution offers the potential for treatment arm stratification by molecular and genomic aberration, and the potential for personalized management options.^19^ For example, a phase 2 trial of Vismodegib, the Focal Adhesion Kinase inhibitor GSK2256098, Capivasertib, and Abemaciclib is currently open to accrual for progressive meningioma harboring specific driver-mutations.^20^ This biomarker-driven trial demonstrates the need for global, multi-institutional efforts to ensure recruitment of a sufficient number of patients from what is a heterogeneous patient pool, into well-defined treatment arms. Repetition of such work, either directly or indirectly, utilizing different outcome measures, could be considered wasteful and should therefore be avoided.^21^

The outcome measures in meningioma clinical trials are not standardized. For example, previous work by Kaley et al.^22^ sought to identify historical outcomes with systemic therapies in order to establish endpoint benchmarks for future clinical trials of medical therapies for recurrent meningioma. This work demonstrated heterogeneity in both the definition of response criteria, and the reported survival outcomes; for instance, some studies reported median overall survival while others reported median PFS. In fact, only PFS at 6 months (PFS-6) was found to be common to all but 1 study in this review. To that end, the importance of standardized outcome reporting was emphasized and PFS-6 was recommended as an outcome to be reported in future studies evaluating interventions for those who have progressed after local therapies (with benchmarks for both WHO grade 1 and WHO grade 2/3 provided), to allow comparative analysis of trial results.^22^ Building on this work, the Response Assessment in Neuro-Oncology Meningioma Working Group published recommendations for assessing response and progression in clinical trials involving patients with meningioma, due to lack of consensus on optimal endpoints, and variation in trial design and response criteria, preventing the comparative analysis of trial results.^23^ While both initiatives highlight concerns regarding outcome measurement and reporting heterogeneity for specific issues (namely progression and response), no initiative has asked what outcomes matter most to key stakeholders (including meningioma patients).

A core outcome set (COS) is defined as the *minimum* set of outcomes that should be measured and reported in all clinical trials for a specific health condition or health area.^24^ COS development is in its infancy within the field of neuro-oncology, but efforts are underway.^25^ The development of COS for meningioma to be used in future clinical effectiveness trials can ensure that the outcomes that are of critical importance to key stakeholders (including meningioma patients), are measured and reported across meningioma clinical studies. Harmonization of outcome measurement and reporting could reduce research waste, and allow meaningful comparison of trial results across similar studies, in order to determine comparative effectiveness. This will be achieved within remit of The COSMIC Project, an international effort to develop 2 COS for meningioma. *COSMIC: Intervention* is being developed for use in phase 2 and later, intracranial meningioma clinical effectiveness trials in adults, that are designed to inform clinical decision-making and improve clinical care for patients. *COSMIC: Observation* is being developed for use in observational clinical studies concerned with incidental, minimally symptomatic, and/or untreated cohorts of patients with intracranial meningioma, that are designed to inform monitoring and decision to treat strategies.^26^

The aim of this systematic review was to identify what outcomes have been measured and reported across meningioma clinical trials and what outcomes are being measured and reported in ongoing studies. The results of this systematic review will be used to inform a long list of outcomes of potential relevance to key stakeholders, including patients with meningioma, which will be prioritized through established consensus methodology to develop the COSMIC: Intervention COS.

## Research Question

What outcomes are measured and reported in ongoing and published clinical trials assessing interventions including surgery, radiotherapy, stereotactic radiosurgery, pharmacotherapy, perioperative care, and supportive treatments, used in isolation or in combination for adult intracranial meningioma?

## Materials and Methods

### Inclusion Criteria

Full-text articles reporting results of phase 2, 3, and 4 clinical trials (including single-arm studies) that assess and report the efficacy of interventions for adult patients with intracranial meningioma were included. Eligible interventions included surgical interventions (including modified techniques, approaches, and adjuncts), fractionated radiotherapy (in any form including conformal 3-dimensional and intensity-modulated radiotherapy), stereotactic radiosurgery (single fraction, hypofractionated or fractionated), pharmacotherapy (whereby the investigators include outcomes related to the effectiveness of the drug, and not simply the tolerability of the drug), perioperative care (including medical therapies, anesthetic considerations, general aspects of the care of patients with intracranial meningioma in and around the time of treatment), and supportive treatments (for example neurorehabilitation and ongoing medical therapies for symptom control). Studies investigating interventions in isolation and in any combination, for example, surgical resection plus a specific radiotherapy and/or chemotherapy regime were included.

A minimum of 20 intracranial meningioma patients per study was required. Patients were adults (18 years and above) of either sex, with a diagnosis of sporadic intracranial meningioma, including multiple meningioma and SMARCE1 loss-related familial meningioma. Histopathological diagnosis was not required, as eligible studies included those where surgical resections were not performed and patients were recruited based on a radiological diagnosis of intracranial meningioma.

Multiple publications relating to the same study were included but considered together, and so repetition of data extraction was not performed (for instance, interim results and subgroup analyses). Studies with a mix of brain tumor types whereby at least 20 patients had an intracranial meningioma were included. Online international trial registries were searched to identify ongoing trials meeting the aforementioned criteria (with an expected accrual greater than 20 patients). Only published trials and online trial registry entries written in the English language were included, due to limitations on resources.

### Exclusion Criteria

Pure safety or experimental studies were not included. Combined-phase studies (for instance, phase 0/2, phase 1/2) were evaluated and discussed between members of the study management group to establish where the focus of the work sat, in order to exclude those with a primary phase 0 or 1 component. Studies were excluded if they included fewer than 20 patients or if they principally described cohorts with spinal meningioma, radiation-induced meningioma (eg, administered in childhood as an intervention for cancer), or associated with the genetic condition NF2-Schwannomatosis.

### Information Sources and Search Strategy

A detailed search strategy utilizing the search strings “meningioma” AND ‘trial’ was developed and translated to interrogate the following electronic bibliographic databases: PubMed, Embase, Medline, CINAHL via EBSCO, and Web of Science. In addition, simple searches of the following trial registries were conducted: Cochrane Central Register of Controlled Trials, ClinicalTrials.gov, and the WHO International Clinical Trials Registry Platform. The search strategies are provided in Supplementary Appendix S1. The searches were first run on April 23, 2020. The searches were re-run on January 24, 2022, to identify new records published since the first search.

### Selection Process

Search results were downloaded from their respective online databases, and uploaded to the online platform Rayyan.^27^ Following de-duplication, 2 review authors (CPM and SMK) independently screened all titles and abstracts that were retrieved, according to the eligibility criteria. Screening was performed on the Rayyan platform independently, and each review author was blind to the screening choices made by the other review author. For titles and abstracts which appeared to meet the eligibility criteria, and for those where a decision could not be confidently made based on title and abstract alone, full-text copies were obtained. All full-text copies were independently screened to assess for eligibility by the same 2 review authors (CPM and SMK). No full-text eligibility checks required escalation to the senior review author (MDJ). The complete reference list of full-text titles included was screened to identify titles not identified through the searches. Trial registry searches were independently performed by a single review author and screened against the same eligibility criteria (CPM) to identify ongoing studies not yet published, which describe outcomes that will be measured and reported.

### Data Items and Data Collection Process

Data were extracted from eligible articles and trial registry entries by a single review author (CPM) into a custom-designed and piloted spreadsheet in Microsoft Excel (v16.34, Microsoft, Washington, DC, USA) following best practice described by the Core Outcome Measures in Effectiveness Trials (COMET) Initiative (The COMET Initiative).^21,24^ The first 10% of included titles were dual extracted by a second review author and confirmed consistency and accuracy of extraction (AII).

The following data was extracted from each study as recommended by COMET^21,24^: Study type, study population, first author, year and journal of publication, intervention(s) under investigation, each outcome reported (recorded verbatim) from the study abstract, methods, or results, the definition of the outcome if provided, whether outcome was a primary or secondary outcome, if stated. The indicator and/or tool(s) used to operationalize or measure the outcome were also extracted when available. The number of verbatim outcomes per trial/study was recorded.

A trial or study outcome is a measurable variable examined in response to a treatment or intervention. An outcome was defined as “one that has original meaning and context.”^28^ Identical outcomes measured at multiple time points were not extracted as different unique outcomes.

### Synthesis Methods

Tabulation and descriptive data analysis were performed in Microsoft Excel (v16.34, Microsoft, Washington, DC, USA) with the aim of deduplicating verbatim outcomes extracted from included studies into a list of unique outcomes, followed by grouping unique outcomes under standardized outcome terms where similar meaning and context exists. Given that there exists considerable heterogeneity in the definition of what constitutes a unique outcome, we utilized the method of data analysis as per Young et al.,^28^ and classify outcomes according to the outcome framework proposed by COMET.^24,29^

### Registration and Protocol

This study is registered with the COMET database as study 1508 and accessible at (https://www.comet-initiative.org/Studies/Details/1508). Institutional review board (University of Liverpool) sponsorship and ethical approval have been obtained for The COSMIC Project (Ref UoL001601).

The review question and question format are summarized in Table 1.

**Table 1.** SDMO (Studies, Data, Methods, and Outcomes) Table Summarizing Review Question and Question Format Structure

Review question	What outcomes are measured and reported in ongoing and published clinical trials assessing the efficacy of interventions for adult intracranial meningioma?
Types of Studies	Published or ongoing phase 2, 3, and 4 clinical trials Minimum of 20 patients recruited or planned.
Types of data	Trial outcomes reported by article and registry authors, that have been measured or plan to be measured, in response to a treatment or intervention.
Types of methods	Choice of outcomes to be measured including outcome definition, method of measurement, and time-point of measurement.
Outcomes	Heterogeneity of outcome measurement and reporting across trials.

## Results

### Studies Identified

**Figure 1.**
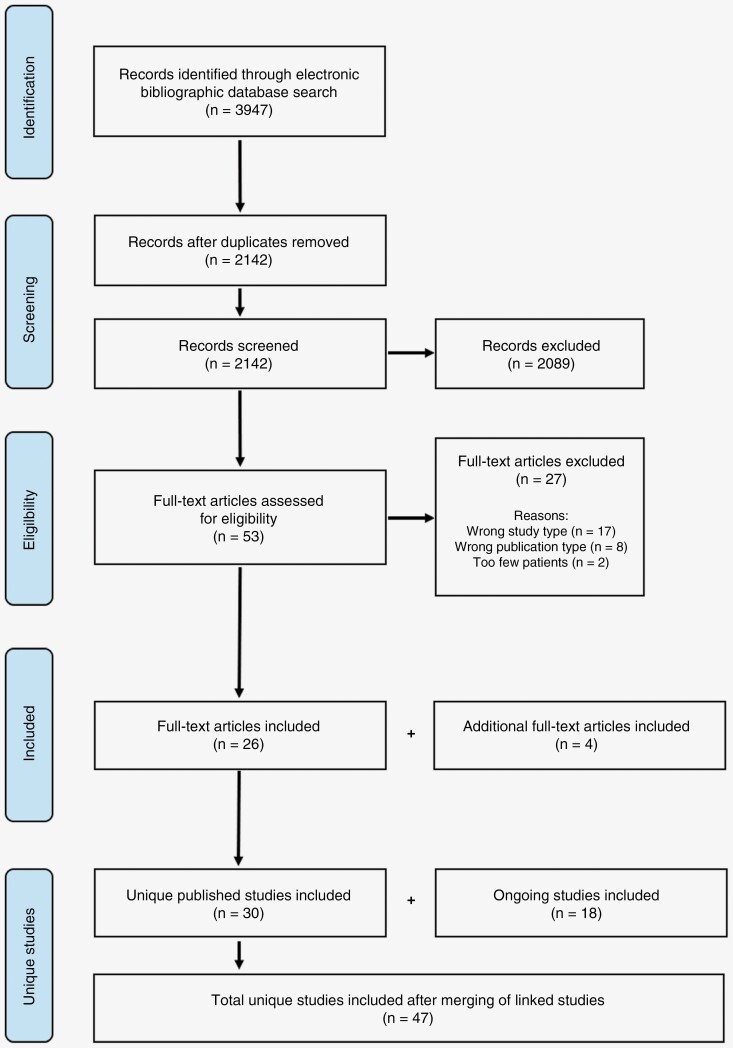
PRISMA flow diagram depicting the identification, screening, eligibility, and inclusion of unique published studies and ongoing studies.

From 3947 records identified following electronic bibliographic database searching, 2142 were screened for inclusion after duplicates were removed, and 53 remained for full-text article eligibility checks. Twenty-seven full-text articles were excluded due to: Wrong study type (*n* = 17), wrong publication type (*n* = 8), and too few patients (*n* = 2). Four additional full-text articles were identified and included following hand-searching of the literature, and 18 ongoing studies were identified in trial registries which were also included. After merging of linked full-texts, 47 unique studies were identified and included in the systematic review (Figure 1). Table 2 shows a summary of the characteristics of the 47 studies (details of the published full-texts are in Supplementary Appendix S2 and ongoing studies in Supplementary Appendix S3).

**Table 2.** Summary of Characteristics of Studies Included in Systematic Review

Characteristic		*N* (No. of studies)
Number of unique studies identified		47
Published		30
Ongoing		18
Year of publication	1990–1999	1
	2000–2009	6
	2010–2019	15
	2020–2022	7
Study phase	II (published)	18
	III (published)	11
	II (ongoing)	15
	III (ongoing)	3
	II (total)	33
	III (total)	14
		***N* (No. of patients)**
Study population	Total No. in systematic review	1611
	Median No. per study (*n* = 29)	42
		***N* (No. of studies)**
Intervention category	Pharmacotherapy	21
	Surgical	13
	Radiotherapy	7
	Radiopeptide therapy	3
	Radiotherapy and pharmacotherapy	3
	Radionuclide therapy	1
	Interstitial therapy	1
		***N* (No. of outcomes)**
Study outcomes	Extracted (published)	576
	Median No. per study (published)	18
	Extracted (ongoing)	83
	Median No. per study (ongoing)	4.5
	Extracted (total)	659
	Median No. per study (total)	10
	With primary outcome designation	40
	With secondary outcome designation	163
	Defined	84

### Outcomes Reported

In total, 659 individual verbatim outcome terms were identified from the 47 included studies. Following de-duplication of identical outcomes (including those with variation in spelling, for example, tumor and tumor), 415 unique verbatim outcome terms remained. A standardized outcome term was selected and applied to each unique verbatim outcome term in order to group those with similar meanings, for example “died of the meningioma” and “died of the disease” were grouped under “meningioma-specific mortality.” Two additional review authors checked the appropriateness and consistency of the standardized outcome terms applied to the unique verbatim outcome terms (AII and MDJ). This resulted in 115 standardized outcome terms. The unique verbatim outcome terms, their frequency of reporting, and the applied standardized outcome terms are listed in Supplementary Appendix S4. The final list of standardized outcome terms, their status as an adverse event or not, their reporting frequency, and the number of those defined within their study of origin are listed in Supplementary Appendix S5.

### Outcome Definitions

Of the 659 individual verbatim outcome terms identified in the included studies, 84 (13%) were accompanied by an outcome definition. Half of these definitions were associated with standardized outcome terms describing progression and survival. Supplementary Appendix S5 shows the reporting frequency of each standardized outcome term, and the individual frequencies of defined standardized outcome terms.

### Mapping of Standardized Outcome Terms to the COMET Taxonomy

Each standardized outcome term was mapped to a COMET outcome domain. In total, 29 domains are represented. The 29 domains map to 5 overarching COMET core areas namely, death, physiological/clinical, life impact, resource use, and adverse events (Supplementary Appendix S5). Table 3 shows the number of studies reporting an individual outcome from each outcome domain, the number of unique outcomes from each domain, and the number of standardized outcome terms from each domain.

**Table 3.** COMET Outcome Domains and Their COMET Core Areas Identified in the Systematic Review

COMET core Area	COMET outcome domain and no.	Studies	Individual outcomes	Unique outcomes	Standardized outcome terms
Death	Mortality/survival (1)	33	98	40	8
Physiological/clinical	Blood and lymphatic system (2)	20	59	36	6
	Cardiac (3)	7	4	4	1
	Endocrine (5)	3	4	4	1
	Ear and labyrinth (6)	4	7	4	2
	Eye outcomes (7)	6	21	19	2
	Gastrointestinal (8)	15	45	20	4
	General outcomes (9)	14	20	6	5
	Hepatobiliary (10)	5	12	8	1
	Immune system (11)	2	2	2	2
	Infection and infestation (12)	6	8	5	3
	Metabolism and nutrition (14)	12	45	27	2
	Musculoskeletal and connective tissue (15)	1	3	3	1
	Nervous system outcomes (17)	43	205	139	53
	Renal and urinary (19)	2	2	2	1
	Reproductive system and breast (20)	2	12	10	1
	Psychiatric (21)	4	8	8	2
	Respiratory, thoracic and mediastinal (22)	2	2	2	1
	Skin and subcutaneous tissue (23)	13	18	11	2
	Vascular (24)	10	12	9	2
Life impact	Functioning (all; 25–29)	4	4	2	1
	Physical functioning (25)	6	6	4	1
	Cognitive functioning (29)	5	7	4	1
	Delivery of care (32)	10	21	20	6
Resource use	Hospital (35)	4	7	7	2
	Need for further intervention (36)	1	1	1	1
Adverse events	Adverse events/effects (38)	24	26	18	3
Total	27	47	659	415	115

COMET, Core Outcome Measures in Effectiveness Trials

#### COMET core area “Death”.—

Eight standardized outcome terms mapped to the COMET outcome domain “mortality/survival” and 33 studies (70%) reported an outcome from this domain. Four of these standardized outcome terms concern binary events, namely “death from pharmacotherapy,” “meningioma-specific mortality,” “overall survival,” and “perioperative mortality,” and 4 were composite outcomes, “further intervention-free survival,” “health-related quality of life” (HRQoL), “deterioration-free survival,” “PFS,” and “recurrence-free survival” (Supplementary Appendix S5). Heterogeneous outcome definitions were identified for “meningioma-specific mortality” (total definitions *n* = 3, unique definitions *n* = 3), “overall survival” (total definitions *n* = 16, unique definitions *n* = 16), and “PFS” (total definitions *n* = 19, unique definitions *n* = 19).

#### COMET core area “Physiological/clinical”.—

The majority of the 115 standardized outcome terms mapped to a COMET outcome domain within the “physical/clinical” COMET core area (*n* = 92, 80%). Out of the 22 “physiological/clinical” outcome domains listed within the COMET taxonomy, nearly all were represented by at least one of the 92 standardized outcome terms (*n* = 19, 86%). Over half of the standardized outcome terms mapped to the COMET outcome domain “nervous system outcomes” (*n* = 53, 58%), and the majority of included studies reported an outcome from this domain (*n* = 43, 91%; Supplementary Appendix S5).

**Figure 2.**
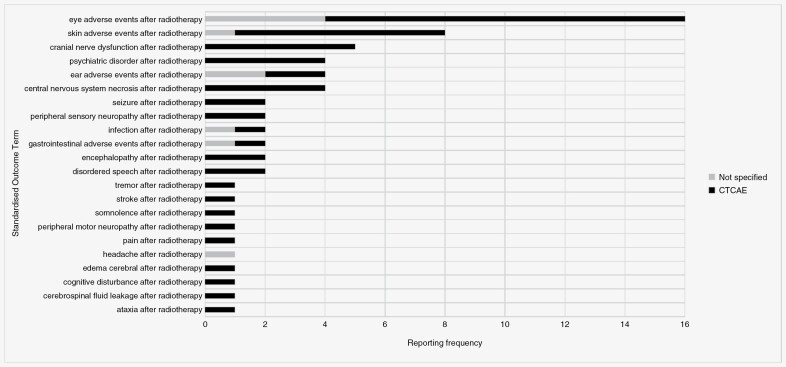
Reporting frequency of radiotherapy-related “adverse outcome” standardized outcome terms, along with the proportion of those with a specified measurement instrument.

**Figure 3.**
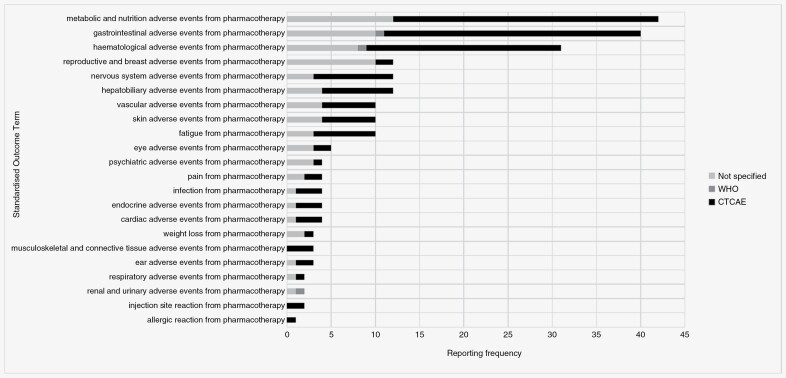
Reporting frequency of pharmacotherapy-related “adverse outcome” standardized outcome terms, along with the proportion of those with a specified measurement instrument.

**Figure 4.**
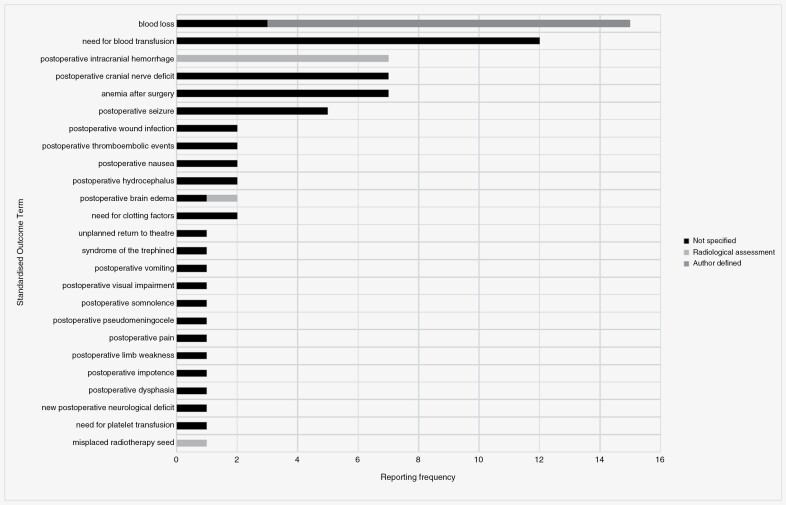
Reporting frequency of surgery-related “adverse outcome” standardized outcome terms, along with the proportion of those with a specified measurement instrument.

Nearly 3-quarters of these “physiological/clinical” standardized outcome terms were also classified as an adverse outcomes (*n* = 68, 74%). Each relates to 1 of the 3 intervention groups, namely radiotherapy (*n* = 22), pharmacotherapy (*n* = 22), or surgery (*n* = 24). The reporting frequency of each, along with the proportion of those with a specified measurement instrument are shown in Figures 2, 3, and 4.

Most of the standardized outcome terms within this COMET core area did not have associated definitions identified (*n* = 82, 89%). Those that were identified were heterogeneous and mostly associated with the following 3 interrelated standardized outcome terms: “complete response” (total definitions *n* = 8, unique definitions *n* = 8), “progressive disease” (total definitions *n* = 10, unique definitions *n* = 10), and “stable disease” (total definitions *n* = 10, unique definitions *n* = 10).

#### COMET core area “Life impact”.—

Nine standardized outcome terms mapped to the “life impact” COMET core area; 3 within the “functioning” COMET outcome domains, and 6 within the “delivery of care” COMET outcome domain. Each is discussed in turn.

The standardized outcome term “health-related quality of life” maps to all 5 “functioning” COMET outcome domains, which includes physical, social, role, emotional, and cognitive functioning, and was reported by 4 studies (9%). Six studies reported the “physical functioning” standardized outcome term which maps to the “physical functioning” COMET outcome domain (13%), and 5 studies reported the “neurocognitive functioning” standardized outcome term which maps to the “cognitive functioning” COMET outcome domain (11%). All 3 of these standardized outcome terms are multidimensional health measures, none of which were defined (Supplementary Appendix S6).

Ten studies reported an outcome from the “delivery of care” COMET outcome domain. Of the 6 “delivery of care” standardized outcome terms, 3 are concerned with deviation from the intended intervention, and are also classified as adverse outcomes. These included “discontinuation of pharmacotherapy due to adverse events,” “discontinuation of radiotherapy due to adverse events,” and reduction of pharmacotherapy dose due to adverse events, none of which were defined. Two standardized outcome terms concerned trial withdrawal “trial withdrawal – Clinician decision” and “trial withdrawal – Patient decision,” and the final standardized outcome term was “unplanned return to theater,” also classified as an adverse outcome. All 6 of these standardized outcome terms are binary events, but only one definition was identified which was for the standardized outcome term “trial withdrawal – Patient decision” (Supplementary Appendix S6).

#### COMET core area “Resource use”.—

Two standardized outcome terms mapped to the COMET outcome domain “hospital,” namely “duration of hospital stay” and “duration of intensive care stay,” and 4 studies (9%) reported an outcome from this domain. The standardized outcome term “need for further treatment” mapped to the COMET outcome domain of the same name and was reported by only one study. Definitions were not identified within this COMET core area. The first 2 can be considered as time-to-event outcomes, while the latter can be considered a binary event (supplementary Appendix S6).

#### COMET core area “Adverse events”.—

Three standardized outcome terms mapped to the COMET outcome domain “adverse events/effects.” Mapping to this COMET core area was reserved for those standardized outcome terms that could not be mapped to a specific “physiological/clinical” COMET outcome domain. Twenty-four studies (51%) reported an outcome from this domain. The 3 standardized outcome terms represent the 3 main intervention categories identified in this review: “adverse events after radiotherapy,” “adverse events from pharmacotherapy,” and “perioperative mortality.” All 3 are clinician-reported multiple-category event standardized outcome terms (Supplementary Appendix S6).

## Discussion

This systematic review has identified 415 unique outcomes measured and reported in 47 published and ongoing meningioma clinical trials. Unique outcomes with the same or similar meaning were grouped together which resulted in the generation of 115 standardized outcome terms. These were classified using the COMET taxonomy into 29 outcome domains and 5 core areas. Most of the standardized outcome terms mapped to the “physiological/clinical” core area, with over half mapping to the domain ‘nervous system outcomes’ specifically. Nearly 3-quarters of these ‘physiological/clinical’ standardized outcome terms were also classified as an adverse outcomes relating to one of surgery, radiotherapy, or pharmacotherapy. The most frequently reported standardized outcome terms were “PFS” and “overall survival,” along with “hematological adverse events from pharmacotherapy,” “gastrointestinal adverse events from pharmacotherapy,” and ‘metabolic and nutritional adverse events from pharmacotherapy’. Over two-thirds of studies included a “mortality/survival” outcome.

This is the first systematic review to identify the breadth of outcomes measured and reported in meningioma clinical effectiveness trials. This has been achieved through the application of a rigorous methodological process described by COMET. We have identified unique outcomes in both published and ongoing phase 2 and 3 trials, across a wide range of interventions including surgery, radiotherapy, and pharmacotherapy. The COMET taxonomy was used to categorize standardized outcome terms, and this was reviewed by experts from the study management and advisory group.

In the introduction to this review, we described previous work by Kaley et al.^22^ that demonstrated heterogeneity in definitions of response criteria and survival, and concluded by stating that PFS at 6 months was common to all but one study analyzed. Our comprehensive analysis of meningioma clinical trials has allowed us to understand the breadth of mortality and time-to-event outcomes selected for measurement by clinical triallists, along with variations in definition (when provided). For example, we applied the standardized outcome term “PFS” to 16 unique outcomes (representing 46 verbatim outcomes) that we considered to have similar “meaning and context.” Some of these unique outcomes differed due to the time-point at which the summary measure was performed, for example, “2-year PFS,” “3-year PFS,” “5-year PFS,” while others differed in name but not application, for example, “PFS rate for 6 months” and “6-month PFS.” As the unique outcome “PFS” was reported most frequently and was most similar in meaning and context to the other 15 unique outcomes under this umbrella, we selected the standardized outcome term “PFS” to represent the 46 identified verbatim outcomes from the literature. Only 19 of the 46 verbatim outcomes were defined within the study from which they came. The absence of a definition allows for misinterpretation of the outcomes meaning and bias when combining results for similar outcomes across trials. Moreover, even when defined, we observed that verbatim outcomes ascribed the same unique outcome term, for example, “progression-free survival” had variable definitions associated with them (6 of the 16 verbatim outcome terms grouped as the unique outcome PFS were defined). Examples of variation in definition for verbatim outcomes we consider to be the same included “From randomization to the first documented disease progression, or death due to any cause, whichever comes first,” “the time from the first day of treatment until disease progression,” and “proportion of patients alive and without progression.”

Adverse events accounted for 77 of the 115 standardized outcome terms applied to the unique outcome identified. As per COMET, we extracted individual adverse events when presented as such, and categorized them within the “physiological/clinical” domain to which they belong where possible. For instance, anemia, leukopenia, and neutropenia were extracted multiple times from studies evaluating pharmacotherapy interventions, but were deduplicated and grouped under the standardized outcome term “hematological adverse events from pharmacotherapy.” We have therefore maintained a degree of granularity when applying standardized outcome terms, which respects both the intervention and “physiological/clinical” area. If “anemia after pharmacotherapy” or simply “anemia” had been selected as the standardized outcome term (and progressed as a potential eDelphi survey item), one could expect that this level of granularity would be too great, of little relevance when developing a COS for this health area, and burdensome to participants during future project stages. Conversely, to have grouped all individual adverse events under a standardized outcome term “adverse events” would be too generic at this stage. The rationalization of standardized outcome terms will be undertaken at a later stage of The COSMIC Project. As surgical adverse events largely mapped to the “nervous system outcomes” domain, we maintained granularity when applying standardized outcome terms, especially as each may have more relevance to participants of the eDelphi survey in later stages of the project. This was also the case for general adverse effects such as “fatigue” and “weight loss”

The process of ascribing a standardized outcome term is of course subjective in itself, but was presented to the study management and study advisory group for consideration for approval. This standardized outcome term can be put forward for rating of importance (along with all other standardized outcome terms selected) by eDelphi survey participants in future stages of this project. In doing so, its inclusion in a COS for meningioma clinical trials would be based on multi-stakeholder consensus. Subsequent work would be undertaken to define such an outcome, to remove heterogeneity in outcome selection and definition/measurement across future trials.

This systematic review has some limitations. The searches were restricted to identify full-texts written only in the English language. This means that there may be studies that report outcomes that we have not identified. However, when the 115 standardized outcome terms were mapped to the COMET taxonomy, 29 outcome domains were represented, thereby demonstrating the breadth of this review. To mitigate against important outcomes that may have been missed, participants recruited to the latter stages of The COSMIC Project will have the opportunity to add new outcomes that they feel are not represented by those in the eDelphi survey. While all search results were dual-screened, data extraction was performed only by the principal investigator for The COSMIC Project (CPM). This was due to financial and personnel limitations. The principal investigator maintained a low threshold for extracting potential unique study outcomes to ensure that all could be considered when data handling was reviewed by members of the study management group. The first 10% of included studies were dual extracted by a second review author (AII) to ensure that data extraction was consistent and accurate, while accepting that what one considers to be a unique outcome is variable.

This systematic review demonstrates that the outcomes measured and reported in meningioma clinical effectiveness trials are numerous, heterogeneous, and poorly and variably defined. The development of a COS for future meningioma clinical trials is therefore justified. The benefit of this could be harmonization of outcome reporting and reduction of research waste for this health area. The standardized outcome terms generated in this systematic review will be rationalized and used to populate a modified eDelphi survey which will be completed by key stakeholders, including patients. We will conduct a one-day consensus meeting of key stakeholders in order to ratify the final COSMIC: Intervention COS. This process of information gathering followed by consensus methodology follows best practices as outlined by COMET. Further work will be required to determine how to measure each core outcome, but data generated from this systematic review on “how” outcomes were measured will provide the basis for this.

## Collaborators

International Consortium on Meningioma: Kenneth Aldape, Abdurrahman I. Islim, Karolyn Au, Jill Barnhartz-Sloan, Wenya Linda Bi, Felix Behling, Priscilla K. Brastianos, Chaya Brodie, Nicholas Butowski, Carlos Carlotti, Ana Castro, Aaron Cohen-Gadol, Marta Couce, Michael D. Cusimano, Francesco DiMeco, Katharine Drummond, Ian F. Dunn, Craig Erker, Michelle Felicella, Daniel M. Fountain, Evanthia Galanis, Norbert Galldiks, Caterina Giannini, Roland Goldbrunner, Brent Griffith, Rintaro Hashizume, C. Oliver Hanemann, Christel Herold-Mende, Luke Hnenny, Craig Horbinski, Raymond Y. Huang, David James, Michael D. Jenkinson, Christine Jungk, Gerhard Jungwirth, Timothy J. Kaufmann, Boris Krischek, Sylvia Kurz, Daniel Lachance, Christian Lafougère, Katrin Lamszus, Ian Lee, Jeff C. Liu, Serge Makarenko, Tathiana Malta, Yasin Mamatjan, Alireza Mansouri, Christian Mawrin, Michael McDermott, Christopher P. Millward, Jennifer Moliterno-Gunel, Andrew Morokoff, David Munoz, Farshad Nassiri, Houtan Noushmehr, Ho-Keung Ng, Arie Perry, Farhad Pirouzmand, Laila M Poisson, Bianca Pollo, Aditya Ragunathan, David R. Raleigh, Mirjam Renovanz, Franz Ricklefs, Felix Sahm, Andrea Saladino, Antonio Santacroce, Thomas Santarius, Jens Schittenhelm, Christian Schichor, David Schultz, Nils O. Schmidt, Warren Selman, Helen Shih, Andrew Sloan, Julian Spears, Matija Snuderl, James Snyder, Suganth Suppiah, Erik Sulman, Ghazaleh Tabatabai, Marcos Tatagiba, Marco Timmer, Daniela Tirapelli, Joerg C. Tonn, Derek Tsang, Michael A. Vogelbaum, Andreas von Deimling, Tobias Walbert, Simon Walling, Justin Z. Wang, Patrick Y. Wen, Manfred Westphal, Adriana M. Workewych, Stephen Yip, Gabriel Zada, Gelareh Zadeh, Viktor Zherebitskiy.

## Supplementary Material

vdae030_suppl_Supplementary_Appendix
