# Supplementary material for: The outcomes measured and reported in intracranial meningioma clinical trials: A systematic review
Source: Neurooncol Adv. 2024 Mar 2;6(1):vdae030. doi: 10.1093/noajnl/vdae030 (PMC11003530; doi:10.1093/noajnl/vdae030)
Supplement: vdae030_suppl_Supplementary_Appendix [file vdae030_suppl_supplementary_appendix.docx]

**Supplementary Appendix 1 - Database and Trial Registry Searches**

**Medline (Ovid)**

| Search | Query |
| --- | --- |
| 1 | meningioma*.tw. |
| 2 | meningioma/ |
| 3 | 1 or 2 |
| 4 | exp clinical trial/ |
| 5 | random allocation/ |
| 6 | double-blind method/ |
| 7 | single-blind method/ |
| 8 | placebos/ |
| 9 | randomized controlled trial.pt. |
| 10 | controlled clinical trial.pt. |
| 11 | clinical trial.pt. |
| 12 | clinical trial, phase ii.pt. |
| 13 | clinical trial, phase iii.pt. |
| 14 | clinical trial, phase iv.pt. |
| 15 | (clin* adj25 trial*).tw. |
| 16 | (control* adj25 trial*).tw. |
| 17 | random*.tw. |
| 18 | ((singl* or doubl* or tripl* or treb*) adj25 (blind* or mask*)).tw. |
| 19 | (phase II or phase 2).tw. |
| 20 | (phase III or phase 3).tw. |
| 21 | (phase IV or phase 4).tw. |
| 22 | placebo*.tw. |
| 23 | 4 or 5 or 6 or 7 or 8 or 9 or 10 or 11 or 12 or 13 or 14 or 15 or 16 or 17 or 18 or 19 or 20 or 21 or 22 |
| 24 | 3 and 23 |
| 25 | exp animals/ not human/ |
| 26 | 24 not 25 |
| 27 | limit 26 to english |

**EMBASE (Ovid)**

| Search | Query |
| --- | --- |
| 1 | ‘meningioma*’:ti,ab |
| 2 | 'meningioma'/de |
| 3 | #1 or #2 |
| 4 | 'clinical trial'/exp |
| 5 | 'randomization'/exp |
| 6 | 'double blind procedure'/de |
| 7 | 'single blind procedure'/de |
| 8 | 'placebo'/de |
| 9 | (clin* NEAR/25 trial*):ti,ab |
| 10 | (control* NEAR/25 trial*):ti,ab |
| 11 | ‘random*’:ti,ab |
| 12 | ((singl* or doubl* or tripl* or treb*) NEAR/25 (blind* or mask*)):ti,ab |
| 13 | ‘phase II’:ti,ab |
| 14 | ‘phase 2’:ti,ab |
| 15 | ‘phase III’:ti,ab |
| 16 | ‘phase 3’:ti,ab |
| 17 | ‘phase IV’:ti,ab |
| 18 | ‘phase 4’:ti,ab |
| 19 | ‘placebo*’:ti,ab |
| 20 | #4 OR #5 OR #6 OR #7 OR #8 OR #9 OR #10 OR #11 OR #12 OR #13 OR #14 OR #15 OR #16 OR #17 OR #18 OR #19 |
| 21 | #3 and #20 |
| 22 | #3 AND #20 AND [english]/lim |

**PubMed**

| Search | Query |
| --- | --- |
| 1 | meningioma*[tiab] |
| 2 | meningioma[mh] |
| 3 | #1 or #2 |
| 4 | clinical trial[mh] |
| 5 | random allocation[mh] |
| 6 | double-blind method[mh] |
| 7 | single-blind method[mh] |
| 8 | placebos[mh] |
| 9 | randomized controlled trial[pt] |
| 10 | controlled clinical trial[pt] |
| 11 | clinical trial[pt] |
| 12 | clinical trial, phase ii[pt] |
| 13 | clinical trial, phase iii[pt] |
| 14 | clinical trial, phase iv[pt] |
| 15 | clinical trial*[tiab] |
| 16 | control trial*[tiab] |
| 17 | controlled trial*[tiab] |
| 18 | random*[tiab] |
| 19 | single blind*[tiab] |
| 20 | double blind*[tiab] |
| 21 | triple blind*[tiab] |
| 22 | treble blind*[tiab] |
| 23 | (phase II[tiab] OR phase 2[tiab]) |
| 24 | (phase III[tiab] OR phase 3[tiab]) |
| 25 | (phase IV[tiab] OR phase 4[tiab]) |
| 26 | placebo*[tiab] |
| 27 | #4 OR #5 OR #6 OR #7 OR #8 OR #9 OR #10 OR #11 OR #12 OR #13 OR #14 OR #15 OR #16 OR #17 OR #18 OR #19 OR #20 OR #21 OR #22 OR #23 OR #24 OR #25 OR #26 |
| 28 | #3 and #27 |
|  | 28 was searched and English language and Human filters were applied |

**CINAHL Plus**

| Search | Query |
| --- | --- |
| S1 | TI meningioma* OR AB meningioma* |
| S2 | MH meningioma |
| S3 | S1 OR S2 |
| S4 | MH "Clinical Trials+" |
| S5 | MH "Random Sample+" |
| S6 | MH "Placebos" |
| S7 | PT randomized controlled trial |
| S8 | PT clinical trial |
| S9 | TI clin* trial* OR AB clin* trial* |
| S10 | TI control* trial* OR AB control* trial* |
| S11 | TI random* OR AB random* |
| S12 | TI ((singl* OR doubl* OR tripl* OR treb*) AND (blind* OR mask*)) |
| S13 | AB ((singl* OR doubl* OR tripl* OR treb*) AND (blind* OR mask*)) |
| S14 | TI (phase II OR phase III OR phase IV OR phase 2 OR phase 3 OR phase 4) |
| S15 | AB (phase II OR phase III OR phase IV OR phase 2 OR phase 3 OR phase 4) |
| S16 | TI placebo* OR AB placebo* |
| S17 | S4 OR S5 OR S6 OR S7 OR S8 OR S9 OR S10 OR S11 OR S12 OR S13 OR S14 OR S15 OR S16 |
| S18 | S3 AND S17 |
| S19 | S3 AND S17: Narrow by Language: - English |

**Web of Science**

| Search | Query |
| --- | --- |
| 1 | TS=(meningioma*) |
| 2 | TS=(clin* NEAR/25 trial*) |
| 3 | TS=(control* NEAR/25 trial*) |
| 4 | TS=(random*) |
| 5 | TS=((singl* or doubl* or tripl* or treb*) NEAR/25 (blind* or mask*)) |
| 6 | TS=(phase II or phase 2) |
| 7 | TS=(phase III or phase 3) |
| 8 | TS=(phase IV or phase 4) |
| 9 | TS=(placebo*) |
| 10 | #2 OR #3 OR #4 OR #5 OR #6 OR #7 OR #8 OR #9 |
| 11 | #1 AND #10 |
| 12 | (#11) AND LANGUAGE: (English) |

**Cochrane central register of controlled trials**

| Search | Query | Notes |
| --- | --- | --- |
| #1 | Meningioma* | Title Abstract Keyword |
| #2 | Meningioma | MeSH term: this term only |
| #3 | #1 OR #2 |  |
| #4 | Clinical trial | MeSH term- explode all trees |
| #5 | Random allocation | MeSH term this term only |
| #6 | Double-blind method | MeSH term this term only |
| #7 | Single-blind method | MeSH term this term only |
| #8 | Placebos | MeSH term this term only |
| #9 | clin* NEAR/25 trial* | Title Abstract Keyword |
| #10 | control* NEAR/25 trial* | Title Abstract Keyword |
| #11 | random* | Title Abstract Keyword |
| #12 | (singl* or doubl* or tripl* or treb*) NEAR/25 (blind* or mask*) | Title Abstract Keyword |
| #13 | phase II OR phase 2 | Title Abstract Keyword |
| #14 | phase III OR phase 3 | Title Abstract Keyword |
| #15 | phase IV OR phase 4 | Title Abstract Keyword |
| #16 | placebo* | Title Abstract Keyword |
| #17 | #4 OR #5 OR #6 OR #7 OR #8 OR #9 OR #10 OR #11 OR #12 OR #13 OR #14 OR #15 OR #16 |  |
| #18 | #3 and #17 |  |
|  | Trials filter selected |  |

**ClinicalTrials.gov**

Search Condition or disease – meningioma

Filters (include) – Recruitment (not yet recruiting, recruiting, enrolling by invitation, active not recruiting, suspended, completed)

Filters (include) – Study type (interventional (clinical trial))

Filters (include) – Study phase (phase 2, phase 3, phase 4)

**WHO International Clinical Trials Registry Platform**

Search – meningioma

Filters (include) – Phases (phase 2, phase 3, phase 4)

**Supplementary Appendix 2 – Studies Included in Systematic Review (Published)**

| **ID** | **Author**  **(year)** | **Study Title** | **Phase** | **Study Population** | **Interventions within study** | **Control or comparator** | **Patients**  **(No./study)** | **Outcomes**  **(No. study)** |
| --- | --- | --- | --- | --- | --- | --- | --- | --- |
| 1 | Vuorinen et al. (1996) | Interstitial radiotherapy of 25 parasellar/clival meningiomas and 19 meningiomas in the elderly. Analysis of short-term tolerance and responses | II | Parasellar/clival location or age ≥ 65 years | Stereotactic implantation of I-125 into intracranial meningiomas | Pretreatment status vs posttreatment status | 44 | 27 |
| 2 | Bendszus et al. (2000) | Efficacy of trisacryl gelatin microspheres versus polyvinyl alcohol particles in the preoperative embolization of meningiomas. | II | Intracranial meningioma | Preoperative embolization of meningioma with trisacryl gelatin microspheres | Preoperative embolization with polyvinyl alcohol particles (2 particle size groups) | 60 | 7 |
| 3 | Hahn et al. (2005) | Prolonged oral hydroxyurea and concurrent 3d-conformal radiation in patients with progressive or recurrent meningioma: results of a pilot study. | II | Recurrent or progressive meningioma | Fractionated 3d-conformal radiation and concurrent Hydroxyurea | Pretreatment status vs posttreatment status | 21 | 27 |
| 4 | Grunberg et al. (2004) | Long-term administration of mifepristone (RU486): clinical tolerance during extended treatment of meningioma. | II | Persistent or recurrent unresectable meningioma | Mifepristone | Pretreatment status vs posttreatment status | 28 | 32 |
| 5 | Chamberlain et al. (2008) | Interferon-alpha for recurrent World Health Organization grade 1 intracranial meningiomas. | II | Recurrent, treatment-refractory, WHO grade 1 meningioma | Interferon-α | Pretreatment status vs posttreatment status | 35 | 22 |
| 6 | Bartolomei et al. (2009) | Peptide receptor radionuclide therapy with (90)Y-DOTATOC in recurrent meningioma. | II | Recurrent or progressive meningioma | Peptide receptor radionuclide therapy with 90Y-DOTATOC | Pretreatment status vs posttreatment status | 29 | 12 |
| 7 | Wen et al. (2009) | Phase II study of imatinib mesylate for recurrent meningiomas (North American Brain Tumor Consortium study 01-08). | II | Histologically confirmed meningioma with unequivocal tumor recurrence | Imatinib | Pretreatment status vs posttreatment status | 22 | 11 |
| 8 | Norden et al. (2010) | Phase II trials of erlotinib or gefitinib in patients with recurrent meningioma. | II | Recurrent histologically confirmed meningiomas with no more than 2 previous chemo regimens | Gefitinib OR Erlotinib | Pretreatment status vs posttreatment status | 25 | 21 |
| 9 | Naqash et al. (2011) | Evaluation of acute normovolemic hemodilution and autotransfusion in neurosurgical patients undergoing excision of intracranial meningioma. | III | Patients undergoing excision of intracranial meningioma | Acute normovolemic hemodilution | Autologous transfusion | 40 | 8 |
| 10 | Reardon et al. (2012) | Phase II study of Gleevec® plus hydroxyurea (HU) in adults with progressive or recurrent meningioma. | II | Progressive or recurrent meningioma | Imatinib and Hydroxyurea | Pretreatment status vs posttreatment status | 21 | 23 |
| 11 | Raizer et al. (2014) | A phase II trial of PTK787/ZK 222584 in recurrent or progressive radiation and surgery refractory meningiomas. | II | Recurrent or progressive radiation and surgery refractory meningioma | Vatalanib | Pretreatment status vs posttreatment status | 25 | 34 |
| 12 | Ji et al. (2015) | Double-Blind Phase III Randomized Trial of the Antiprogestin Agent Mifepristone in the Treatment of Unresectable Meningioma: SWOG S9005. | III | Primary, recurrent, or residual unresectable meningioma | Mifepristone | Placebo | 164 | 18 |
| 13 | Marincek et al. (2015) | Somatostatin receptor-targeted radiopeptide therapy with 90Y-DOTATOC and 177Lu-DOTATOC in progressive meningioma: long-term results of a phase II clinical trial. | II | Progressive unresectable meningioma | 90Y-DOTATOC and 177Lu-DOTATOC | Pretreatment status vs posttreatment status | 34 | 5 |
| 14 | Kaley et al. (2015) | Phase II trial of sunitinib for recurrent and progressive atypical and anaplastic meningioma. | II | Surgery and radiation-refractory recurrent WHO grades II-III meningioma | Sunitinib | Pretreatment status vs posttreatment status | 36 | 43 |
| 15 | Norden et al. (2015) | Phase II study of monthly pasireotide LAR (SOM230C) for recurrent or progressive meningioma. | II | Histologically confirmed recurrent or progressive meningioma of any grade | Pasireotide LAR | Pretreatment status vs posttreatment status | 34 | 48 |
| 16 | Yang et al. (2017) | Evaluation of acute normovolemic hemodilution in patients undergoing intracranial meningioma resection: A quasi-experimental trial. | III | Patients undergoing excision of intracranial meningioma | Acute normovolemic hemodilution and intraoperative cell salvage | Intraoperative cell salvage | 80 | 14 |
| 17 | Sanford et al. (2017) | Prospective, Randomized Study of Radiation Dose Escalation With Combined Proton-Photon Therapy for Benign Meningiomas. | III | Patients with incompletely resected or recurrent benign meningioma | Fractionated combined proton-photon radiotherapy at an escalated dose (63.0 Gy) | Lower combined proton-photon radiotherapy dose (55.8 Gy) | 44 | 10 |
| 18 | Hooda et al. (2017) | Effect of tranexamic acid on intraoperative blood loss and transfusion requirements in patients undergoing excision of intracranial meningioma. | III | Patients undergoing surgery for intracranial meningioma | Tranexamic acid | Placebo | 60 | 26 |
| 19 | Iacobucci et al. (2017) | Preoperative embolization of meningiomas with polyvinyl alcohol particles: The benefits are not outweighed by risks. | III | Patients requiring surgical resection and significant blood loss expected | Preoperative embolization of meningioma with polyvinyl alcohol particles | Surgery alone | 134 | 6 |
| 20 | Weber et al. (2018) | Adjuvant postoperative high-dose radiotherapy for atypical and malignant meningioma: A phase-II parallel non-randomized and observation study (EORTC 22042-26042). | II | Patients with atypical meningioma (WHO grade II) and Simpson's grade 1-3 | High dose radiation therapy (60 Gy) | Pretreatment status vs posttreatment status | 56 | 43 |
| 21 | Hegazy et al. (2018) | Mobilization of the outer cavernous membrane decreases bleeding and improves resection in spheno-clinoidal meningiomas without cavernous sinus extension: A randomized controlled trial. | III | Patients with spheno-clinoidal meningioma without cavernous sinus involvement | Mobilization of the outer cavernous sinus membrane | Direct opening of the dura without cavernous sinus mobilization | 94 | 12 |
| 22a | Rogers et al. (2018) | Intermediate-risk meningioma: initial outcomes from NRG Oncology RTOG 0539. | II | Newly diagnosed WHO Grade II menignioma treated with gross-total resection (GTR; Simpson Grades I-III) or recurrent WHO Grade I meningioma with any resection extent. | Radiation therapy, either intensity modulated, 3D conformal, or proton therapy; (54 Gy in 30 fractions) | Historical controls (3-year PFS: 70% following GTR alone and 90% with GTR + RT) | 52 | 28 |
| 22b | Rogers et al. (2020) | High-risk Meningioma: Initial Outcomes FromFfrom NRG Oncology/RTOG 0539. | II | Newly diagnosed or recurrent WHO grade III meningioma of any resection extent, a recurrent WHO grade II meningioma of any resection extent, or a newly diagnosed WHO grade II meningioma after STR | Intensity-modulated radiation therapy using simultaneous integrated boost; (60 Gy in 30 fractions) | Historical controls (3-year PFS: 50% following surgery and RT) | 57 | N/A |
| 23 | Siddiqui et al. (2018) | Use of tranexamic acid to reduce intraoperative bleeding in craniotomy for meningioma patients | III | Patients undergoing surgery for intracranial meningioma | Tranexamic acid | Placebo | 100 | 11 |
| 24 | Graillon et al. (2020) | Everolimus and Octreotide for Patients with Recurrent Meningioma: Results from the Phase II CEVOREM Trial. | II | Recurrent tumor progression ineligible for further surgery/radiotherapy | Everolimus and Octreotide LAR | Pretreatment status vs posttreatment status | 20 | 20 |
| 25 | Premkumar et al. (2020) | Effect of intravenous magnesium sulphate on patients undergoing craniotomy for meningioma excision: A randomised controlled study | III | Patients undergoing surgery for supratentorial meningioma excision | Magnesium sulphate | Placebo | 60 | 7 |
| 26 | Ravi et al. (2021) | Effect of tranexamic acid on blood loss, coagulation profile, and quality of surgical field in intracranial meningioma resection: A prospective randomized, double-blind, placebo-controlled study | III | Patients undergoing elective meningioma resection surgery | Tranexamic acid | Placebo | 30 | 6 |
| 27 | Rebar et al. (2021) | Intraoperative tranexamic acid use in patients undergoing excision of intracranial meningioma: Randomized, placebo-controlled trial | III | Patients scheduled to undergo excision of intracranial meningioma | Tranexamic acid | Placebo | 91 | 17 |
| 28 | Preusser et al. (2021) | Trabectedin for recurrent WHO grade 2 or 3 meningioma: a randomized phase 2 study of the EORTC Brain Tumor Group (EORTC-1320-BTG) | II | Patients with recurrent WHO grade 2 or 3 meningioma | Trabectedin | Local standard of care | 90 | 11 |
| 29 | Linda Bi et al. (2022) | Activity of PD-1 blockade with nivolumab among patients with recurrent atypical/anaplastic meningioma: phase II trial results | II | Patients with grade 2 or 3 meningioma that recurred after surgery and radiation therapy | Nivolumab | Pretreatment status vs posttreatment status | 25 | 27 |

**Supplementary Appendix 3 – Studies Included in Systematic Review (Ongoing)**

| **ID** | **Chief Investigator**  **Registration No.** | **Study Title** | **Phase** | **Study Population** | **Interventions within study** | **Control or comparator** | **Study**  **Status** | **Outcomes**  **(No. study)** |
| --- | --- | --- | --- | --- | --- | --- | --- | --- |
| 30 | Scott Randall Plotkin  NCT03071874 | A Single Arm Phase II Study Of The Dual mTORC1/mTORC2 Inhibitor Vistusertib (AZD2014) Provided On An Intermittent Schedule For Sporadic Patients With Grade II-III Meningiomas That Recur Or Progress After Surgery And Radiation | II | Patients with histologically confirmed intracranial meningioma, grade II-III,that has recurred or progressed at previous treatment | Vistusertib | Pretreatment status vs posttreatment status | Active, not recruiting | 5 |
| 31 | David Reardon  NCT02648997  (arm 2 of study 29) | An Open-Label Phase II Study of Nivolumab in Adult Participants With Recurrent High-Grade Meningioma | II | Patients with grade 2 or 3 meningioma that recurred after surgery and radiation therapy | Radiation therapy (IMRT, 3D-CRT, or proton-beam) and Nivolumab and Ipilimumab | Pretreatment status vs posttreatment status | Recruiting | 7 |
| 32 | Walter Stummer & Bernard Bendok  NCT04305470 | A Phase 3 Multicenter Study of Gleolan (Aminolevulinic Acid Hydrochloride) to Enhance Visualization of Tumor in Patients With Newly Diagnosed or Recurrent Meningiomas | III | Patients about to undergo resection for suspected meningioma | 5-ALA | Uncontrolled | Recruiting | 3 |
| 33 | Priscilla Brastianos  NCT03279692 | Phase II Trial of Pembrolizumab in Recurrent or Residual High Grade Meningioma | II | Histologically proven recurrent or residual intracranial or metastatic meningioma or meningioma with extracranial spread | Pembrolizumab | Pretreatment status vs posttreatment status | Active, not recruiting | 4 |
| 34 | Erik Sukman &  Sylvia Kurz  NCT03971461 | A Single Arm, Open-label, Multicenter Phase II Study of 177Lu-DOTATATE Radionuclide in Adults With Progressive or High-risk Meningioma | II | Patients with progressive WHO I-III or residual high-risk Ga-DOTATATE PET-MRI positive meningioma | 177Lu-DOTATATE | Pretreatment status vs posttreatment status | Recruiting | 5 |
| 35 | Lode J. Swinnen & Geoffrey R. Barger  NCT00003590 | Phase II Study of Hydroxyurea for Unresectable Meningioma | II | Histologically confirmed unresectable primary, recurrent, or residual benign meningioma | Hydroxyurea | Pretreatment status vs posttreatment status | Completed (not published) | 2 |
| 36 | C. Leland Rogers  NCT03180268 | Phase III Trial of Observation Versus Irradiation for a Gross Totally Resected Grade II Meningioma | III | Newly diagnosed unifocal intracranial meningioma, gross totally resected, and histologically confirmed as WHO grade II based upon pathology findings at the enrolling institution; (WHO 2016 criteria) | Radiation therapy (59.4 Gy in 33 daily fractions of 1.8 Gy each) | Observation | Recruiting | 11 |
| 37 | Priscilla Brastianos  NCT02523014 | Phase II Trial of SMO/ AKT/ NF2/CDK Inhibitors in Progressive Meningiomas With SMO/ AKT/ NF2/CDK Pathway Mutations | II | Patients with meningioma that is growing, spreading, or getting worse (progressive) | Vismodegib or FAK Inhibitor GSK2256098 or Capivasertib or Abemaciclib | Pretreatment status vs posttreatment status | Recruiting | 4 |
| 38 | Shlomit Yust-Katz  NCT03016091 | A Phase II, Open-label, Single Arm Trial of Pembrolizumab for Refractory Atypical and Anaplastic Meningioma | II | Recurrent or progressive meningioma (WHO grades II-III) | Pembrolizumab | Pretreatment status vs posttreatment status | Recruiting | 2 |
| 39 | Nancy Ann  Oberheim Bush  NCT04659811 | A Phase II Study of Stereotactic Radiosurgery in Conjunction With the PD-1 Inhibitor, Pembrolizumab for the Treatment of Recurrent Meningioma | II | Participants with recurrent grade II or III meningioma or Participants with multiple recurrent (>=2) grade I meningioma | Stereotactic radiosurgery and Pembrolizumab | Historical control based on progression free survival at 12 months (PFS12) | Recruiting | 5 |
| 40 | Kenneth W. Merrell  NCT04082520 | A Prospective, Phase II Study of Lutetium Lu 177 Dotatate (LUTATHERA®) in Patients With Inoperable, Progressive Meningioma After External Beam Radiation Therapy | II | Patients with meningioma that cannot be treated with surgery (inoperable) and is growing, spreading, or getting worse (progressive) after external beam radiation therapy | 177Lu-DOTATATE | Pretreatment status vs posttreatment status | Recruiting | 7 |
| 41 | Priya Kumthekar  NCT01125046 | Phase II Trial of Bevacizumab in Patients With Recurrent or Progressive Meningiomas | II | Patients With Recurrent or Progressive Meningioma | Bevacizumab | Pretreatment status vs posttreatment status | Completed (not published) | 3 |
| 42 | Juergen Debus  ISRCTN58692836 | Treatment of Patients With Atypical Meningiomas Simpson Grade 4 and 5 With a Carbon Ion Boost in Combination With Postoperative Photon Radiotherapy: A Phase II Trial | II | Patients with atypical meningiomas after incomplete resection or biopsy | Carbon ion radiotherapy (Carbon Ion Boost 18 Gy E in single Fractions of 3 Gy E) | Pretreatment status vs posttreatment status | Recruiting | 2 |
| 43 | Michael D. Jenkinson  ISRCTN71502099 | Radiation versus observation following surgical resection of atypical meningioma | III | Newly diagnosed unifocal intracranial meningioma, gross totally resected, and histologically confirmed as WHO grade II based upon pathology findings at the enrolling institution; (WHO 2016 criteria) | Radiation therapy (59.4 Gy in 33 daily fractions of 1.8 Gy each) | Observation | Active, not recruiting | 6 |
| 44 | Kohmura Eiji  JPRN-UMIN000031156 | Phase II study of photodynamic therapy (PDT) for malignant meningioma | II | Patients with grade 2 or 3 meningioma | Photodynamic therapy | Pretreatment status vs posttreatment status | Recruiting | 2 |
| 45 | Vijay M Patil CTRI/2019/02/017499 | A phase 2 study of Gemcitabine In recurrent Meningioma for evaluation of Efficacy | II | Patients with recurrent meningioma grade 2 or 3, who are not amenable for local therapy and are planned for palliative intent systemic therapy | Gemcitabine | Pretreatment status vs posttreatment status | Recruiting | 6 |
| 46 | Dai Kamamoto  JPRN-jRCT2031190074 | Anti-PD-1 antibody therapy for recurrent / progressive Meningioma: an investigator-initiated, open-label, single-arm, multicenter, phase II clinical trial | II | Patients with recurrent or progressive meningioma | Nivolumab | Pretreatment status vs posttreatment status | Recruiting | 6 |
| 47 | Masahide Matsuda  JPRN-jRCTs031210158 | A Prospective Interventional Study to Evaluate the Efficacy and Safety of Intraoperative Fluorescence Diagnosis Using 5-ALA in Meningioma surgery | II | Patients undergoing surgery for meningioma | 5-ALA | Uncontrolled | Recruiting | 3 |

**Supplementary Appendix 4 – Standardised outcome terms applied to unique verbatim outcome terms**

| **Unique verbatim outcome term** | **Reporting frequency** | **Standardised outcome term applied** |
| --- | --- | --- |
| tumor growth rates | 1 | absolute growth rate |
| acute and late adverse events | 1 | adverse events after radiotherapy |
| adverse Events | 1 | adverse events after radiotherapy |
| safety | 1 | adverse events after radiotherapy |
| toxicities | 1 | adverse events after radiotherapy |
| adverse events of radiotherapy | 1 | adverse events after radiotherapy |
| adverse events | 4 | adverse events from pharmacotherapy |
| overall safety | 1 | adverse events from pharmacotherapy |
| safety | 3 | adverse events from pharmacotherapy |
| Safety and tolerability | 1 | adverse events from pharmacotherapy |
| safety profile | 1 | adverse events from pharmacotherapy |
| tolerability | 1 | adverse events from pharmacotherapy |
| adverse events | 3 | adverse events from pharmacotherapy |
| treatment related adverse events | 1 | adverse events from pharmacotherapy |
| allergic reaction | 1 | allergic reaction from pharmacotherapy |
| Hb (during various stages of surgery) | 3 | anemia after surgery |
| changes in hemoglobin levels | 1 | anemia after surgery |
| final hematocrit | 1 | anemia after surgery |
| haematocrit (at maximum blood loss) | 2 | anemia after surgery |
| ataxia | 1 | ataxia after radiotherapy |
| blood lost | 1 | blood loss |
| bleeding in the intraoperative and (postoperative) period | 1 | blood loss |
| estimated blood loss | 2 | blood loss |
| intraoperative blood loss | 6 | blood loss |
| post operative blood loss | 1 | blood loss |
| postoperative blood loss | 1 | blood loss |
| surgical blood loss | 1 | blood loss |
| total blood loss | 2 | blood loss |
| myocardial infarction | 1 | cardiac adverse events from pharmacotherapy |
| prolonged QTc interval | 1 | cardiac adverse events from pharmacotherapy |
| right ventricular enlargement | 1 | cardiac adverse events from pharmacotherapy |
| sinus bradycardia | 1 | cardiac adverse events from pharmacotherapy |
| atrophy (of CNS tissue) | 1 | central nervous system necrosis after radiotherapy |
| brain necrosis | 1 | central nervous system necrosis after radiotherapy |
| cerebral necrosis | 1 | central nervous system necrosis after radiotherapy |
| cns necrosis | 1 | central nervous system necrosis after radiotherapy |
| leak, cerebrospinal fluid | 1 | cerebrospinal fluid leakage after radiotherapy |
| prothrombin time (PT) | 2 | clotting function after surgery |
| activated partial thromboplastin time (APTT) | 1 | clotting function after surgery |
| coagulation profile | 1 | clotting function after surgery |
| fibrinogen | 1 | clotting function after surgery |
| platelet amount | 1 | clotting function after surgery |
| cognitive disturbance | 1 | cognitive disturbance after radiotherapy |
| complete response | 10 | complete response |
| tumor regression | 1 | complete response |
| tumor response | 1 | complete response |
| tumor volume reduction rate | 1 | complete response |
| olfactory nerve disorder | 1 | cranial nerve dysfunction after radiotherapy |
| trigeminal nerve disorder | 1 | cranial nerve dysfunction after radiotherapy |
| neuropathy cranial CN II Vision | 1 | cranial nerve dysfunction after radiotherapy |
| neuropathy cranial CN V motor-jaw muscles; sensory-facial | 1 | cranial nerve dysfunction after radiotherapy |
| neuropathy cranial CN VII motor-face; sensory-taste | 1 | cranial nerve dysfunction after radiotherapy |
| deaths (attributable to αIFN) | 1 | death from pharmacotherapy |
| visualization to identify tissue as likely or unlikely to be meningioma among indeterminate tissues | 1 | diagnostic efficacy of 5-ALA during surgery |
| gleolan-induced PpIX fluorescence among indeterminate tissue and unexpected fluorescent EOS tissue locations | 1 | diagnostic efficacy of 5-ALA during surgery |
| tumor positive diagnosis rate of additionally removed specimens | 1 | diagnostic efficacy of 5-ALA during surgery |
| discontinued study therapy due to adverse events | 1 | discontinuation of pharmacotherapy due to adverse events |
| discontinued treatment | 1 | discontinuation of pharmacotherapy due to adverse events |
| off study because of adverse events | 1 | discontinuation of pharmacotherapy due to adverse events |
| physician decision | 1 | discontinuation of pharmacotherapy due to adverse events |
| removed due to adverse events | 1 | discontinuation of pharmacotherapy due to adverse events |
| unacceptable adverse events | 1 | discontinuation of pharmacotherapy due to adverse events |
| withdrawal for adverse events | 1 | discontinuation of pharmacotherapy due to adverse events |
| interrupted RT | 1 | discontinuation of radiotherapy due to adverse events |
| prematurely stopped RT | 1 | discontinuation of radiotherapy due to adverse events |
| speech disorder | 1 | disordered speech after radiotherapy |
| speech impairment | 1 | disordered speech after radiotherapy |
| distribution of the embolic agent | 1 | distribution of embolization agent |
| duration of ICU and (hospital) stay | 1 | duration of hospital stay |
| length of hospital stay | 1 | duration of hospital stay |
| postoperative hospital stay | 1 | duration of hospital stay |
| postoperative stay in hospital | 1 | duration of hospital stay |
| duration of (ICU) and hospital stay | 1 | duration of intensive care stay |
| duration of post-operative ventilation | 1 | duration of intensive care stay |
| ICU stay | 1 | duration of intensive care stay |
| duration of anesthesia | 1 | duration of surgery |
| duration of surgery | 3 | duration of surgery |
| operative time | 1 | duration of surgery |
| hearing loss | 1 | ear adverse events after radiotherapy |
| vertigo | 1 | ear adverse events after radiotherapy |
| dizziness | 2 | ear adverse events after radiotherapy |
| dizziness | 3 | ear adverse events from pharmacotherapy |
| cerebral oedema | 1 | edema cerebral after radiotherapy |
| electrolytes | 1 | electrolyte status after surgery |
| magnesium levels | 1 | electrolyte status after surgery |
| signs of hypermagnesemia | 1 | electrolyte status after surgery |
| encephalopathy | 1 | encephalopathy after radiotherapy |
| leukoencephalopathy | 1 | encephalopathy after radiotherapy |
| bone pain | 1 | endocrine adverse events from pharmacotherapy |
| endocrine disorders—other | 1 | endocrine adverse events from pharmacotherapy |
| hyperthyroidism | 1 | endocrine adverse events from pharmacotherapy |
| hypothyroidism | 1 | endocrine adverse events from pharmacotherapy |
| extent of tumor resection | 1 | extent of resection |
| additional tumor removal | 1 | extent of resection |
| extent of tumor removal | 1 | extent of resection |
| tumor resection | 1 | extent of resection |
| amaurosis | 1 | eye adverse events after radiotherapy |
| blurred vision | 2 | eye adverse events after radiotherapy |
| cataract | 1 | eye adverse events after radiotherapy |
| decline of vision | 1 | eye adverse events after radiotherapy |
| diplopia | 1 | eye adverse events after radiotherapy |
| dry eye | 1 | eye adverse events after radiotherapy |
| dry eye syndrome | 2 | eye adverse events after radiotherapy |
| flashing vision | 1 | eye adverse events after radiotherapy |
| glaucoma | 1 | eye adverse events after radiotherapy |
| keratitis | 1 | eye adverse events after radiotherapy |
| photophobia | 1 | eye adverse events after radiotherapy |
| retinopathy | 1 | eye adverse events after radiotherapy |
| visual loss | 1 | eye adverse events after radiotherapy |
| watery eye | 1 | eye adverse events after radiotherapy |
| extraocular muscle function | 1 | eye adverse events from pharmacotherapy |
| optic nerve disorder | 1 | eye adverse events from pharmacotherapy |
| uveitis | 1 | eye adverse events from pharmacotherapy |
| visual accomodation | 1 | eye adverse events from pharmacotherapy |
| visual field | 1 | eye adverse events from pharmacotherapy |
| fatigue | 10 | fatigue from pharmacotherapy |
| time to second line (salvage) treatment | 1 | further intervention-free survival |
| gastrointestinal symtpoms | 1 | gastrointestinal adverse events after radiotherapy |
| nausea | 1 | gastrointestinal adverse events after radiotherapy |
| abdominal distension/bloating | 1 | gastrointestinal adverse events from pharmacotherapy |
| abdominal pain | 4 | gastrointestinal adverse events from pharmacotherapy |
| constipation | 2 | gastrointestinal adverse events from pharmacotherapy |
| diarrhea | 6 | gastrointestinal adverse events from pharmacotherapy |
| duodenal ulcer | 1 | gastrointestinal adverse events from pharmacotherapy |
| dysgeusia | 1 | gastrointestinal adverse events from pharmacotherapy |
| dyspepsia | 1 | gastrointestinal adverse events from pharmacotherapy |
| flatulence | 1 | gastrointestinal adverse events from pharmacotherapy |
| gastrointestinal perforation | 1 | gastrointestinal adverse events from pharmacotherapy |
| gastrointestinal adverse events | 1 | gastrointestinal adverse events from pharmacotherapy |
| GI | 2 | gastrointestinal adverse events from pharmacotherapy |
| incontinence, anal | 1 | gastrointestinal adverse events from pharmacotherapy |
| mucositis (oral) | 1 | gastrointestinal adverse events from pharmacotherapy |
| nausea | 10 | gastrointestinal adverse events from pharmacotherapy |
| stomatitis | 2 | gastrointestinal adverse events from pharmacotherapy |
| vomiting | 5 | gastrointestinal adverse events from pharmacotherapy |
| anemia | 4 | haematological adverse events from pharmacotherapy |
| granulocytopenia | 1 | haematological adverse events from pharmacotherapy |
| hematological adverse events | 1 | haematological adverse events from pharmacotherapy |
| hemoglobin | 2 | haematological adverse events from pharmacotherapy |
| leukocytes | 1 | haematological adverse events from pharmacotherapy |
| leukopenia | 6 | haematological adverse events from pharmacotherapy |
| lymphocytopenia | 2 | haematological adverse events from pharmacotherapy |
| minimal leucocyte-counts | 1 | haematological adverse events from pharmacotherapy |
| minimum Hb-levels | 1 | haematological adverse events from pharmacotherapy |
| neutropenia | 4 | haematological adverse events from pharmacotherapy |
| platelets | 1 | haematological adverse events from pharmacotherapy |
| thrombocytopenia | 6 | haematological adverse events from pharmacotherapy |
| white blood cell adverse events | 1 | haematological adverse events from pharmacotherapy |
| quality of surgical field during resection of intracranial meningioma | 1 | haemostasis of surgical field during surgery |
| haemostasis at the time of surgery | 1 | haemostasis of surgical field during surgery |
| quality of surgical hemostasis | 1 | haemostasis of surgical field during surgery |
| headache | 1 | headache after radiotherapy |
| health-related QoL | 1 | health-related quality of life |
| quality of life | 3 | health-related quality of life |
| ALT | 2 | hepatobillary adverse events from pharmacotherapy |
| amylase | 1 | hepatobillary adverse events from pharmacotherapy |
| AST | 3 | hepatobillary adverse events from pharmacotherapy |
| cholelithiasis | 1 | hepatobillary adverse events from pharmacotherapy |
| elevated serum glutamic pyruvic transaminase | 1 | hepatobillary adverse events from pharmacotherapy |
| GGT | 1 | hepatobillary adverse events from pharmacotherapy |
| pancreatitis | 2 | hepatobillary adverse events from pharmacotherapy |
| transaminase | 1 | hepatobillary adverse events from pharmacotherapy |
| time to deterioration in QOL | 1 | HRQoL deterioration-free survival |
| Circulating Immune Cell Subsets and Cytokines | 1 | immunological response to pharmacotherapy |
| acute external otitis | 1 | infection after radiotherapy |
| infection | 1 | infection after radiotherapy |
| infection | 4 | infection from pharmacotherapy |
| infusion-related reaction | 1 | injection site reaction from pharmacotherapy |
| injection site reaction | 1 | injection site reaction from pharmacotherapy |
| intracranial haemorrhage | 1 | intracranial hemorrhage |
| intratumoural bleeding | 1 | intracranial hemorrhage |
| occult blood | 1 | intracranial hemorrhage |
| serious bleeding | 1 | intracranial hemorrhage |
| tumour bleeding | 1 | intracranial hemorrhage |
| tumour size … during follow-up | 1 | maximum 2D size of the tumor |
| 3 Year Disease-Specific Survival (DSS) | 1 | meningioma-specific mortality |
| 5 Year Disease-Specific Survival (DSS) | 1 | meningioma-specific mortality |
| actuarial survival rate | 1 | meningioma-specific mortality |
| died | 1 | meningioma-specific mortality |
| died of meningioma | 1 | meningioma-specific mortality |
| died of the disease | 1 | meningioma-specific mortality |
| disease-related deaths | 1 | meningioma-specific mortality |
| disease-Specific Survival (DSS) | 1 | meningioma-specific mortality |
| alkaline phosphatase | 1 | metabolic and nutrition adverse events from pharmacotherapy |
| anorexia | 2 | metabolic and nutrition adverse events from pharmacotherapy |
| biochemical adverse events | 1 | metabolic and nutrition adverse events from pharmacotherapy |
| CPK | 1 | metabolic and nutrition adverse events from pharmacotherapy |
| creatinine | 1 | metabolic and nutrition adverse events from pharmacotherapy |
| dehydration | 4 | metabolic and nutrition adverse events from pharmacotherapy |
| hyperammonemia | 1 | metabolic and nutrition adverse events from pharmacotherapy |
| hypercalcemia | 2 | metabolic and nutrition adverse events from pharmacotherapy |
| hypercholesterolemia | 2 | metabolic and nutrition adverse events from pharmacotherapy |
| hyperglycemia | 5 | metabolic and nutrition adverse events from pharmacotherapy |
| hypertriglyceridemia | 2 | metabolic and nutrition adverse events from pharmacotherapy |
| hyperuricemia | 1 | metabolic and nutrition adverse events from pharmacotherapy |
| hypoalbuminemia | 2 | metabolic and nutrition adverse events from pharmacotherapy |
| hypocalcemia | 1 | metabolic and nutrition adverse events from pharmacotherapy |
| hypoglycemia | 1 | metabolic and nutrition adverse events from pharmacotherapy |
| hypokalemia | 2 | metabolic and nutrition adverse events from pharmacotherapy |
| hypomagnesemia | 1 | metabolic and nutrition adverse events from pharmacotherapy |
| hyponatremia | 2 | metabolic and nutrition adverse events from pharmacotherapy |
| hypophosphatemia | 4 | metabolic and nutrition adverse events from pharmacotherapy |
| lipase | 2 | metabolic and nutrition adverse events from pharmacotherapy |
| poor oral intake | 1 | metabolic and nutrition adverse events from pharmacotherapy |
| proteinuria | 1 | metabolic and nutrition adverse events from pharmacotherapy |
| Serum amylase increased | 1 | metabolic and nutrition adverse events from pharmacotherapy |
| SGPT elevation | 1 | metabolic and nutrition adverse events from pharmacotherapy |
| minor response | 2 | minor response |
| misplaced seed | 1 | misplaced radiotherapy seed |
| arthritis | 1 | musculoskeletal and connective tissue adverse events from pharmacotherapy |
| muscle pain | 1 | musculoskeletal and connective tissue adverse events from pharmacotherapy |
| muscle weakness | 1 | musculoskeletal and connective tissue adverse events from pharmacotherapy |
| blood transfusion | 2 | need for blood transfusion |
| intra-operative PRBC transfusion | 1 | need for blood transfusion |
| cell saver blood | 1 | need for blood transfusion |
| amount of blood transfused | 1 | need for blood transfusion |
| blood transfusion requirement | 1 | need for blood transfusion |
| transfusion requirements | 1 | need for blood transfusion |
| homologous blood transfused | 1 | need for blood transfusion |
| post-operative PRBC transfusion | 1 | need for blood transfusion |
| volume of homologous blood transfused | 1 | need for blood transfusion |
| amount of autologous blood withdrawn and retransfused | 1 | need for blood transfusion |
| intra-operative use of cell saver | 1 | need for blood transfusion |
| fresh frozen plasma (transfusion) | 1 | need for clotting factors |
| frozen plasma transfused | 1 | need for clotting factors |
| re-resection and systematic therapy | 1 | need for further intervention |
| platelets (transfusion) | 1 | need for platelet transfusion |
| ataxia | 1 | nervous system adverse events from pharmacotherapy |
| CNS hemorrhage | 2 | nervous system adverse events from pharmacotherapy |
| confusion | 1 | nervous system adverse events from pharmacotherapy |
| edema cerebral | 1 | nervous system adverse events from pharmacotherapy |
| encephalopathy | 1 | nervous system adverse events from pharmacotherapy |
| headache | 4 | nervous system adverse events from pharmacotherapy |
| sensation | 1 | nervous system adverse events from pharmacotherapy |
| weakness (motor neuropathy) | 1 | nervous system adverse events from pharmacotherapy |
| cognition | 1 | neurocognitive functioning |
| memory impairment | 2 | neurocognitive functioning |
| mental status | 1 | neurocognitive functioning |
| neurocognitive function | 3 | neurocognitive functioning |
| progressive neurological deficit | 1 | neurologic status after treatment |
| neurologic function | 2 | neurologic status after treatment |
| neurological progression | 1 | neurologic status after treatment |
| Patient Reported Outcomes (PRO) | 1 | neurologic symptom burden after treatment |
| subsequent course of pre-operative symptoms | 1 | neurologic symptom burden after treatment |
| new neurologic deficit | 1 | new postoperative neurological deficit |
| 12-month survival rates | 1 | overall survival |
| 2-year OS | 1 | overall survival |
| 3-year overall survival | 2 | overall survival |
| 5 Year Overall Survival (OS) | 1 | overall survival |
| 6-month survival rates | 1 | overall survival |
| OS at 6 months | 1 | overall survival |
| overall survival | 27 | overall survival |
| overall survival at 12 months (OS-12) | 2 | overall survival |
| survival | 1 | overall survival |
| pain | 1 | pain after radiotherapy |
| pain | 4 | pain from pharmacotherapy |
| partial response | 1 | partial response |
| perioperative complications | 2 | perioperative adverse events |
| adverse events | 1 | perioperative adverse events |
| complications | 1 | perioperative adverse events |
| safety | 1 | perioperative adverse events |
| study safety assessment | 1 | perioperative adverse events |
| postoperative death | 1 | perioperative mortality |
| post-operative mortality within 48 hours | 1 | perioperative mortality |
| neuropathy motor | 1 | peripheral motor neuropathy after radiotherapy |
| neuropathy sensory | 1 | peripheral sensory neuropathy after radiotherapy |
| peripheral sensory neuropathy | 1 | peripheral sensory neuropathy after radiotherapy |
| neurmuscular blockers usage | 1 | pharmacotherapy requirements during surgery |
| colloids | 1 | pharmacotherapy requirements during surgery |
| crystalloids | 1 | pharmacotherapy requirements during surgery |
| intra-operative opioid requirement | 1 | pharmacotherapy requirements during surgery |
| functional outcomes | 1 | physical functioning |
| KPS | 2 | physical functioning |
| neurological outcome | 2 | physical functioning |
| performance status | 1 | physical functioning |
| heart rate | 4 | physiological response to surgery |
| baseline temperature | 1 | physiological response to surgery |
| CVP | 2 | physiological response to surgery |
| mean arterial pressure | 4 | physiological response to surgery |
| tumor swelling | 1 | postoperative brain edema |
| oedema | 1 | postoperative brain edema |
| cranial nerve palsy | 1 | postoperative cranial nerve deficit |
| cranial nerve deficits | 1 | postoperative cranial nerve deficit |
| cranial nerve injury | 1 | postoperative cranial nerve deficit |
| cranial nerve palsies | 1 | postoperative cranial nerve deficit |
| facial nerve palsy | 1 | postoperative cranial nerve deficit |
| facial numbness | 1 | postoperative cranial nerve deficit |
| injury to the III cranial nerve | 1 | postoperative cranial nerve deficit |
| weakness or dysphasia | 1 | postoperative dysphasia |
| hydrocephalus | 2 | postoperative hydrocephalus |
| partial impotence | 1 | postoperative impotence |
| hematoma formation | 2 | postoperative intracranial hemorrhage |
| hemiparesis | 1 | postoperative limb weakness |
| nausea | 2 | postoperative nausea |
| brief pain | 1 | postoperative pain |
| cerebrospinal fluid collection | 1 | postoperative pseudomeningocele |
| convulsive seizure | 1 | postoperative seizure |
| early seizure incidence | 1 | postoperative seizure |
| epilepsy | 1 | postoperative seizure |
| seizer activity | 1 | postoperative seizure |
| seizures | 1 | postoperative seizure |
| oversedated | 1 | postoperative somnolence |
| thromboembolic complications | 1 | postoperative thromboembolic events |
| thrombotic complications | 1 | postoperative thromboembolic events |
| aggravation of visual deficit | 1 | postoperative visual impairment |
| vomiting | 1 | postoperative vomiting |
| postoperative infection | 1 | postoperative wound infection |
| wound infection | 1 | postoperative wound infection |
| 12-months PFS rate (PFS12) | 1 | progression-free survival |
| 2-year PFS | 1 | progression-free survival |
| 3-year PFS | 3 | progression-free survival |
| 5-year PFS | 2 | progression-free survival |
| 6-month progression-free survival rate (PFS6) | 1 | progression-free survival |
| disease free survival [DFS] | 1 | progression-free survival |
| duration of local control | 1 | progression-free survival |
| duration of radiographic Response | 1 | progression-free survival |
| failure free survival | 1 | progression-free survival |
| PFS at 12 months | 2 | progression-free survival |
| progression free survival | 16 | progression-free survival |
| progression free time | 1 | progression-free survival |
| progression-free survival at 6 months | 9 | progression-free survival |
| progression-free survival rate for 6 months | 1 | progression-free survival |
| six-month progression-free survival | 4 | progression-free survival |
| time to progression | 1 | progression-free survival |
| disease progression | 1 | progressive disease |
| progressive disease | 9 | progressive disease |
| tumor progression | 9 | progressive disease |
| decreased libido | 1 | psychiatric adverse events from pharmacotherapy |
| depression | 1 | psychiatric adverse events from pharmacotherapy |
| mood or conscious change | 1 | psychiatric adverse events from pharmacotherapy |
| mood swings/depression | 1 | psychiatric adverse events from pharmacotherapy |
| anxiety | 1 | psychiatric disorder after radiotherapy |
| agitation | 1 | psychiatric disorder after radiotherapy |
| confusion | 1 | psychiatric disorder after radiotherapy |
| depression | 1 | psychiatric disorder after radiotherapy |
| best objective response | 1 | radiographic response to treatment |
| best overall response | 1 | radiographic response to treatment |
| Confirmed and Unconfirmed Complete Response (CR) or Partial Response (PR) | 1 | radiographic response to treatment |
| Intracranial response | 1 | radiographic response to treatment |
| objective radiographic response rate | 2 | radiographic response to treatment |
| Objective Radiologic Response Rate | 1 | radiographic response to treatment |
| objective response rate | 2 | radiographic response to treatment |
| objective tumor response rate | 1 | radiographic response to treatment |
| overall response | 1 | radiographic response to treatment |
| radiographic response | 1 | radiographic response to treatment |
| radiographic response rate | 3 | radiographic response to treatment |
| response rate | 3 | radiographic response to treatment |
| response to treatment | 1 | radiographic response to treatment |
| extent of devascularization | 1 | radiological response to embolization |
| extent of necrosis | 1 | radiological response to embolization |
| inflammatory reaction | 1 | radiological response to embolization |
| coagulation necrosis | 1 | radiological response to interstitial radiotherapy |
| hypodense rings | 1 | radiological response to interstitial radiotherapy |
| liquefied necrotic cysts | 1 | radiological response to interstitial radiotherapy |
| shrunk | 1 | radiological response to interstitial radiotherapy |
| time to local recurrence | 1 | recurrence-free survival |
| 3-year actuarial local failure rate | 1 | recurrence-free survival |
| dose modification | 2 | reduction of pharmacotherapy dose due to adverse events |
| dose reduction (as a consequence of adverse events) | 1 | reduction of pharmacotherapy dose due to adverse events |
| relative tumour volume | 1 | relative growth rate |
| 3d tumor growth rate | 1 | relative growth rate |
| GU | 1 | renal and urinary adverse events from pharmacotherapy |
| renal adverse events | 1 | renal and urinary adverse events from pharmacotherapy |
| atrophic endometrial polyps | 1 | reproductive and breast adverse events from pharmacotherapy |
| benign ovarian serous cystadenoma | 1 | reproductive and breast adverse events from pharmacotherapy |
| cessation of menses | 1 | reproductive and breast adverse events from pharmacotherapy |
| endometrial hyperplasia | 1 | reproductive and breast adverse events from pharmacotherapy |
| endometrial polyp | 1 | reproductive and breast adverse events from pharmacotherapy |
| gynecomastia | 2 | reproductive and breast adverse events from pharmacotherapy |
| menses change | 1 | reproductive and breast adverse events from pharmacotherapy |
| peritoneal adenocarcinoma | 1 | reproductive and breast adverse events from pharmacotherapy |
| thickened endometrium | 1 | reproductive and breast adverse events from pharmacotherapy |
| vaginal bleeding | 2 | reproductive and breast adverse events from pharmacotherapy |
| pneumonitis | 1 | respiratory adverse events from pharmacotherapy |
| pneumopathy | 1 | respiratory adverse events from pharmacotherapy |
| seizure | 2 | seizure after radiotherapy |
| alopezia | 1 | skin adverse events after radiotherapy |
| dermatitis radiation | 1 | skin adverse events after radiotherapy |
| hyperpigmentation | 1 | skin adverse events after radiotherapy |
| hypopigmentation | 1 | skin adverse events after radiotherapy |
| pruritus | 1 | skin adverse events after radiotherapy |
| rash | 1 | skin adverse events after radiotherapy |
| skin complaints | 1 | skin adverse events after radiotherapy |
| ulceration | 1 | skin adverse events after radiotherapy |
| alopecia | 3 | skin adverse events from pharmacotherapy |
| dry skin | 1 | skin adverse events from pharmacotherapy |
| rash | 6 | skin adverse events from pharmacotherapy |
| somnolence/depressed level of consciousness | 1 | somnolence after radiotherapy |
| continual no evidence of disease | 1 | stable disease |
| disease control | 1 | stable disease |
| local control rate | 1 | stable disease |
| stable disease | 11 | stable disease |
| stable disease or no response | 1 | stable disease |
| CVA | 1 | stroke after radiotherapy |
| post-craniotomy syndrome | 1 | syndrome of the trephined |
| tremor | 1 | tremor after radiotherapy |
| came off study due to progressive disease | 1 | trial withdrawal - Clinician decision |
| protocol violation | 1 | trial withdrawal - Clinician decision |
| elected to discontinue study therapy | 1 | trial withdrawal - Patient decision |
| self-discontinued chemotherapy | 1 | trial withdrawal - Patient decision |
| voluntary discontinuance | 1 | trial withdrawal - Patient decision |
| withdrawal of consent to pursue alternative therapy | 1 | trial withdrawal - Patient decision |
| withdrew | 1 | trial withdrawal - Patient decision |
| withdrew consent | 1 | trial withdrawal - Patient decision |
| re-exploration | 1 | unplanned return to theatre |
| DVT | 2 | vascular adverse events from pharmacotherapy |
| edema | 1 | vascular adverse events from pharmacotherapy |
| hot flashes | 2 | vascular adverse events from pharmacotherapy |
| hypertension | 2 | vascular adverse events from pharmacotherapy |
| pedal edema | 1 | vascular adverse events from pharmacotherapy |
| thrombophlebitis | 1 | vascular adverse events from pharmacotherapy |
| thrombotic microangiopathy | 1 | vascular adverse events from pharmacotherapy |
| CT volumes | 1 | volume of tumor |
| cuboidal tumor volume | 1 | volume of tumor |
| weight loss | 3 | weight loss from pharmacotherapy |
| **415** | **659** | **115 (unique standardised outcome terms)** |

**Supplementary Appendix 5 – Standardised outcome terms and reporting frequency**

| **COMET outcome area** | **COMET outcome domain** | **Standardised outcome term** | **Adverse event?** | **Reporting frequency** | **No. defined** |
| --- | --- | --- | --- | --- | --- |
| Death | Mortality/survival (1) | death from pharmacotherapy | AE | 1 | 0 |
|  |  | further intervention-free survival |  | 1 | 1 |
|  |  | HRQoL deterioration-free survival |  | 1 | 0 |
|  |  | meningioma-specific mortality |  | 8 | 3 |
|  |  | overall survival |  | 37 | 16 |
|  |  | perioperative mortality | AE | 2 | 0 |
|  |  | progression-free survival |  | 46 | 19 |
|  |  | recurrence-free survival |  | 2 | 0 |
| Physiological/clinical | Blood & lymphatic system (2) | anemia after surgery | AE | 7 | 1 |
|  |  | clotting function after surgery |  | 6 | 0 |
|  |  | haematological adverse events from pharmacotherapy | AE | 31 | 0 |
|  |  | need for blood transfusion | AE | 12 | 2 |
|  |  | need for clotting factors | AE | 2 | 0 |
|  |  | need for platelet transfusion | AE | 1 | 0 |
|  | Cardiac (3) | cardiac adverse events from pharmacotherapy | AE | 4 | 0 |
|  | Endocrine (5) | endocrine adverse events from pharmacotherapy | AE | 4 | 0 |
|  | Ear & labyrinth (6) | ear adverse events after radiotherapy | AE | 4 | 0 |
|  |  | ear adverse events from pharmacotherapy | AE | 3 | 0 |
|  | Eye (7) | eye adverse events after radiotherapy | AE | 16 | 0 |
|  |  | eye adverse events from pharmacotherapy | AE | 5 | 1 |
|  | Gastrointestinal (8) | gastrointestinal adverse events after radiotherapy | AE | 2 | 0 |
|  |  | gastrointestinal adverse events from pharmacotherapy | AE | 40 | 0 |
|  |  | postoperative nausea | AE | 2 | 0 |
|  |  | postoperative vomiting | AE | 1 | 0 |
|  | General (9) | fatigue from pharmacotherapy | AE | 10 | 0 |
|  |  | injection site reaction from pharmacotherapy | AE | 2 | 0 |
|  |  | pain after radiotherapy | AE | 1 | 0 |
|  |  | pain from pharmacotherapy | AE | 4 | 0 |
|  |  | weight loss from pharmacotherapy | AE | 3 | 0 |
|  | Hepatobilliary (10) | hepatobillary adverse events from pharmacotherapy | AE | 12 | 0 |
|  | Immune system (11) | allergic reaction from pharmacotherapy | AE | 1 | 0 |
|  |  | immunological response to pharmacotherapy |  | 1 | 0 |
|  | Infection & infestation (12) | infection after radiotherapy | AE | 2 | 0 |
|  |  | infection from pharmacotherapy | AE | 4 | 0 |
|  |  | postoperative wound infection | AE | 2 | 0 |
|  | Metabolism & nutrition (14) | electrolyte status after surgery |  | 3 | 0 |
|  |  | metabolic and nutrition adverse events from pharmacotherapy | AE | 42 | 0 |
|  | Musculoskeletal & connective tissue (15) | musculoskeletal and connective tissue adverse events from pharmacotherapy | AE | 3 | 0 |
|  | Nervous system (17) | absolute growth rate |  | 1 | 0 |
|  |  | ataxia after radiotherapy | AE | 1 | 0 |
|  |  | blood loss | AE | 15 | 3 |
|  |  | central nervous system necrosis after radiotherapy | AE | 4 | 0 |
|  |  | cerebrospinal fluid leakage after radiotherapy | AE | 1 | 0 |
|  |  | cognitive disturbance after radiotherapy | AE | 1 | 0 |
|  |  | complete response |  | 13 | 8 |
|  |  | cranial nerve dysfunction after radiotherapy | AE | 5 | 0 |
|  |  | diagnostic efficacy of 5-ALA during surgery |  | 3 | 0 |
|  |  | disordered speech after radiotherapy | AE | 2 | 0 |
|  |  | distribution of embolization agent |  | 1 | 0 |
|  |  | duration of surgery |  | 5 | 0 |
|  |  | edema cerebral after radiotherapy | AE | 1 | 0 |
|  |  | encephalopathy after radiotherapy | AE | 2 | 0 |
|  |  | extent of resection |  | 4 | 0 |
|  |  | haemostasis of surgical field during surgery |  | 3 | 0 |
|  |  | headache after radiotherapy | AE | 1 | 0 |
|  |  | maximum 2D size of the tumor |  | 1 | 0 |
|  |  | minor response |  | 2 | 0 |
|  |  | misplaced radiotherapy seed | AE | 1 | 0 |
|  |  | nervous system adverse events from pharmacotherapy | AE | 12 | 0 |
|  |  | neurologic status after treatment |  | 4 | 1 |
|  |  | neurologic symptom burden after treatment |  | 2 | 0 |
|  |  | new postoperative neurological deficit | AE | 1 | 0 |
|  |  | partial response |  | 1 | 0 |
|  |  | peripheral motor neuropathy after radiotherapy | AE | 1 | 0 |
|  |  | peripheral sensory neuropathy after radiotherapy | AE | 2 | 0 |
|  |  | pharmacotherapy requirements during surgery |  | 4 | 1 |
|  |  | physiological response to surgery |  | 11 | 0 |
|  |  | postoperative brain edema | AE | 2 | 0 |
|  |  | postoperative cranial nerve deficit | AE | 7 | 0 |
|  |  | postoperative dysphasia | AE | 1 | 0 |
|  |  | postoperative hydrocephalus | AE | 2 | 0 |
|  |  | postoperative impotence | AE | 1 | 0 |
|  |  | postoperative intracranial hemorrhage | AE | 7 | 0 |
|  |  | postoperative limb weakness | AE | 1 | 0 |
|  |  | postoperative pain | AE | 1 | 0 |
|  |  | postoperative pseudomeningocele | AE | 1 | 0 |
|  |  | postoperative seizure | AE | 5 | 0 |
|  |  | postoperative somnolence | AE | 1 | 0 |
|  |  | postoperative visual impairment | AE | 1 | 0 |
|  |  | progressive disease |  | 19 | 10 |
|  |  | radiographic response to treatment |  | 19 | 2 |
|  |  | radiological response to embolization |  | 3 | 0 |
|  |  | radiological response to interstitial radiotherapy |  | 4 | 0 |
|  |  | relative growth rate |  | 2 | 0 |
|  |  | seizure after radiotherapy | AE | 2 | 0 |
|  |  | somnolence after radiotherapy | AE | 1 | 0 |
|  |  | stable disease |  | 15 | 10 |
|  |  | stroke after radiotherapy | AE | 1 | 0 |
|  |  | syndrome of the trephined | AE | 1 | 0 |
|  |  | tremor after radiotherapy | AE | 1 | 0 |
|  |  | volume of tumor |  | 2 | 0 |
|  | Renal and urinary (19) | renal and urinary adverse events from pharmacotherapy | AE | 2 | 0 |
|  | Reproductive system & breast (20) | reproductive and breast adverse events from pharmacotherapy | AE | 12 | 0 |
|  | Psychiatric (21) | psychiatric adverse events from pharmacotherapy | AE | 4 | 0 |
|  |  | psychiatric disorder after radiotherapy | AE | 4 | 0 |
|  | Respiratory, thoracic & mediastinal (22) | respiratory adverse events from pharmacotherapy | AE | 2 | 0 |
|  | Skin & subcutaneous tissue (23) | skin adverse events after radiotherapy | AE | 8 | 0 |
|  |  | skin adverse events from pharmacotherapy | AE | 10 | 0 |
|  | Vascular (24) | postoperative thromboembolic events | AE | 2 | 0 |
|  |  | vascular adverse events from pharmacotherapy | AE | 10 | 0 |
| Life impact | Functioning (all) (25-29) | health-related quality of life |  | 4 | 0 |
|  | Physical functioning (25) | physical functioning |  | 6 | 0 |
|  | Cognitive functioning (29) | neurocognitive functioning |  | 7 | 0 |
|  | Delivery of care (32) | discontinuation of pharmacotherapy due to adverse events | AE | 7 | 0 |
|  |  | discontinuation of radiotherapy due to adverse events | AE | 2 | 0 |
|  |  | reduction of pharmacotherapy dose due to adverse events | AE | 3 | 0 |
|  |  | trial withdrawal - Clinician decision |  | 2 | 0 |
|  |  | trial withdrawal - Patient decision |  | 6 | 1 |
|  |  | unplanned return to theatre | AE | 1 | 0 |
| Resource use | Hospital (35) | duration of hospital stay |  | 4 | 0 |
|  |  | duration of intensive care stay |  | 3 | 0 |
|  | Need for further intervention (36) | need for further intervention |  | 1 | 0 |
| Adverse events | Adverse events/effects (38) | adverse events after radiotherapy | AE | 5 | 1 |
|  |  | adverse events from pharmacotherapy | AE | 15 | 4 |
|  |  | perioperative adverse events | AE | 6 | 0 |
|  | **29** | **115** | **77** | **659** | **84** |

**Supplementary Appendix 6 – Types of standardised outcome terms identified**

| **COMET Core Area** | **Binary event/time to event** | **Composite** | **Multiple category event**  **Clinician Reported** | **Multiple category event**  **Patient Reported** | **Multi-dimensional**  **health measures** |
| --- | --- | --- | --- | --- | --- |
| ***Death*** | death from pharmacotherapy | further intervention-free survival |  |  |  |
|  | meningioma-specific mortality | progression-free survival |  |  |  |
|  | overall survival | recurrence-free survival |  |  |  |
|  | Perioperative mortality | HRQoL deterioration-free survival |  |  |  |
| ***Physiological/clinical*** |  |  | anemia after surgery |  |  |
|  |  |  | clotting function after surgery |  |  |
|  |  |  | haematological adverse events from pharmacotherapy |  |  |
|  |  |  | need for blood transfusion |  |  |
|  |  |  | need for clotting factors |  |  |
|  |  |  | need for platelet transfusion |  |  |
|  |  |  | cardiac adverse events from pharmacotherapy |  |  |
|  |  |  | endocrine adverse events from pharmacotherapy |  |  |
|  |  |  | ear adverse events from pharmacotherapy |  |  |
|  |  |  | ear adverse events after radiotherapy |  |  |
|  |  |  | eye adverse events after radiotherapy |  |  |
|  |  |  | eye adverse events from pharmacotherapy |  |  |
|  |  |  | gastrointestinal adverse events after radiotherapy |  |  |
|  |  |  | gastrointestinal adverse events from pharmacotherapy |  |  |
|  |  |  | postoperative nausea |  |  |
|  |  |  | postoperative vomiting |  |  |
|  |  |  | fatigue from pharmacotherapy |  |  |
|  |  |  | injection site reaction from pharmacotherapy |  |  |
|  |  |  | pain after radiotherapy |  |  |
|  |  |  | pain from pharmacotherapy |  |  |
|  |  |  | weight loss from pharmacotherapy |  |  |
|  |  |  | hepatobiliary adverse events from pharmacotherapy |  |  |
|  |  |  | allergic reaction from pharmacotherapy |  |  |
|  |  |  | immunological response to pharmacotherapy |  |  |
|  |  |  | infection after radiotherapy |  |  |
|  |  |  | infection from pharmacotherapy |  |  |
|  |  |  | postoperative wound infection |  |  |
|  |  |  | electrolyte status after surgery |  |  |
|  |  |  | metabolic and nutrition adverse events from pharmacotherapy |  |  |
|  |  |  | musculoskeletal and connective tissue adverse events from pharmacotherapy |  |  |
|  |  |  | blood loss |  |  |
|  |  |  | central nervous system necrosis after radiotherapy |  |  |
|  |  |  | cerebrospinal fluid leakage after radiotherapy |  |  |
|  |  |  | cognitive disturbance after radiotherapy |  |  |
|  |  |  | complete response |  |  |
|  |  |  | cranial nerve dysfunction after radiotherapy |  |  |
|  |  |  | diagnostic efficacy of 5-ALA during surgery |  |  |
|  |  |  | disordered speech after radiotherapy |  |  |
|  |  |  | distribution of embolization agent |  |  |
|  |  |  | duration of surgery |  |  |
|  |  |  | edema cerebral after radiotherapy |  |  |
|  |  |  | encephalopathy after radiotherapy |  |  |
|  |  |  | extent of resection |  |  |
|  |  |  | haemostasis of surgical field during surgery |  |  |
|  |  |  | headache after radiotherapy |  |  |
|  |  |  | maximum 2D size of the tumor |  |  |
|  |  |  | minor response |  |  |
|  |  |  | misplaced radiotherapy seed |  |  |
|  |  |  | nervous system adverse events from pharmacotherapy |  |  |
|  |  |  | neurologic status after treatment |  |  |
|  |  |  | new postoperative neurological deficit |  |  |
|  |  |  | partial response |  |  |
|  |  |  | peripheral motor neuropathy after radiotherapy |  |  |
|  |  |  | peripheral sensory neuropathy after radiotherapy |  |  |
|  |  |  | pharmacotherapy requirements during surgery |  |  |
|  |  |  | physiological response to surgery |  |  |
|  |  |  | postoperative brain edema |  |  |
|  |  |  | postoperative cranial nerve deficit |  |  |
|  |  |  | postoperative dysphasia |  |  |
|  |  |  | postoperative hydrocephalus |  |  |
|  |  |  | postoperative impotence |  |  |
|  |  |  | postoperative intracranial hemorrhage |  |  |
|  |  |  | postoperative limb weakness |  |  |
|  |  |  | postoperative pain |  |  |
|  |  |  | postoperative pseudomeningocele |  |  |
|  |  |  | postoperative seizure |  |  |
|  |  |  | postoperative somnolence |  |  |
|  |  |  | postoperative visual impairment |  |  |
|  |  |  | progressive disease |  |  |
|  |  |  | radiographic response to treatment |  |  |
|  |  |  | radiological response to embolization |  |  |
|  |  |  | radiological response to interstitial radiotherapy |  |  |
|  |  |  | relative growth rate |  |  |
|  |  |  | seizure after radiotherapy |  |  |
|  |  |  | somnolence after radiotherapy |  |  |
|  |  |  | stable disease |  |  |
|  |  |  | stroke after radiotherapy |  |  |
|  |  |  | syndrome of the trephined |  |  |
|  |  |  | tremor after radiotherapy |  |  |
|  |  |  | volume of tumor |  |  |
|  |  |  | renal and urinary adverse events from pharmacotherapy |  |  |
|  |  |  | reproductive and breast adverse events from pharmacotherapy |  |  |
|  |  |  | psychiatric adverse events from pharmacotherapy |  |  |
|  |  |  | psychiatric disorder after radiotherapy |  |  |
|  |  |  | respiratory adverse events from pharmacotherapy |  |  |
|  |  |  | skin adverse events after radiotherapy |  |  |
|  |  |  | skin adverse events from pharmacotherapy |  |  |
|  |  |  | postoperative thromboembolic events |  |  |
|  |  |  | vascular adverse events from pharmacotherapy |  |  |
| ***Life impact*** |  |  |  |  | neurologic symptom burden after treatment |
|  |  |  |  |  | health-related quality of life |
|  |  |  |  |  | physical functioning |
|  |  |  |  |  | neurocognitive functioning |
| ***Resource use*** |  |  | duration of hospital stay |  |  |
|  |  |  | duration of intensive care stay |  |  |
| ***Adverse events*** |  |  | adverse events after radiotherapy |  |  |
|  |  |  | adverse events from pharmacotherapy |  |  |
|  |  |  | perioperative adverse events |  |  |
